# Optical Enantiodifferentiation of Chiral Nitriles

**DOI:** 10.1021/acs.orglett.4c02758

**Published:** 2024-09-04

**Authors:** Jeffrey S. S. K. Formen, Christian Wolf

**Affiliations:** Chemistry Department, Georgetown University, Washington D.C. 20057, United States

## Abstract

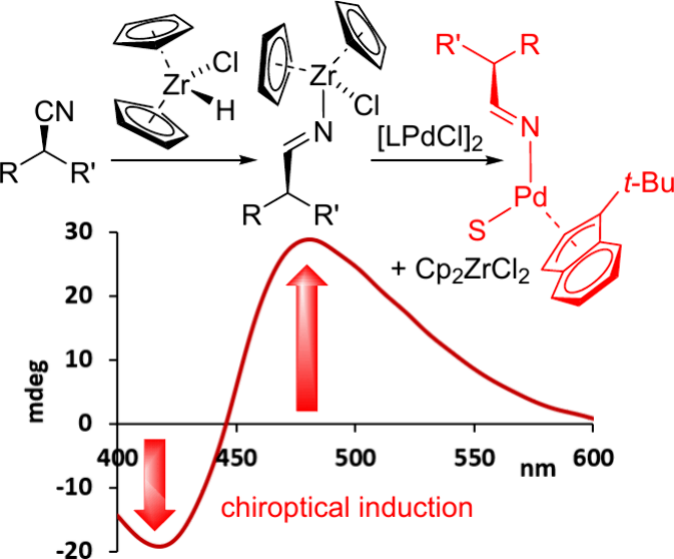

Chiroptical sensing of nitriles is achieved with excellent functional group tolerance by hydrozirconation and subsequent transmetalation of the corresponding iminate to a chromophoric palladium complex. A one-pot workflow that uses the Schwartz reagent and [(η^3^-1-*tert*-butylindenyl)(μ-Cl)Pd]_2_ as sensor generates a palladium complex displaying red-shifted CD inductions and characteristic UV changes. These chiroptical responses are accurately correlated to the enantiomeric ratio and total concentration of the original nitrile.

Chiral nitriles are attractive building blocks with widespread use in organic synthesis and an important structural component in natural products and pharmaceuticals including cyanocycline A, pregnenolone 16 α-carbonitrile, vildagliptin, saxagliptin, and alogliptin. The versatility of nitriles, which may serve as precursors of various other functionalities such as amines, aldehydes, ketones, amides, carboxylic acids, and *N*-heterocycles, makes them a popular choice in asymmetric synthesis, and the incorporation of a cyano group, for example, as a metabolically stable bioisostere of a carbonyl or halogen moiety, has become a viable drug development strategy.^1^ Not surprisingly, the broad utility and general significance of chiral nitriles have received considerable attention and inspired the development of asymmetric methods that provide access to a large variety of enantioenriched structures.^2−11^ By contrast, the stereochemical analysis of chiral nitriles is routinely restricted to traditional enantioselective chromatography,^12,13^ although sensing of the absolute configuration of cyanohydrins^14^ and elegant NMR methods that rely on the formation of nontransient diastereomeric adducts^15,16^ have also been reported.

The current dependence on HPLC, GC, and NMR methods, which are inherently serial techniques, that is, they follow laborious, time-consuming workflows by analyzing one sample at a time, imposes critical high-throughput screening limitations. To overcome these and other shortcomings, intriguing alternatives based on mass spectrometry,^17^ UV,^18^ fluorescence,^19−21^ gas-phase rotational resonance,^22^ IR,^23^ electronic circular dichroism (ECD),^24^ fluorescence-detected CD spectroscopy,^25^ and biochemical methods^26^ have been introduced. Among these advances, chiroptical sensing methods which are compatible with separation-free high-throughput experimentation equipment and allow parallel analysis of hundreds of samples using automated liquid dispensing and multiwell plate technologies have probably been most impactful.^27−29^ To date, the optical sensing field has largely seen the development of probes that bind amines, amino alcohols, amino acids, diols, and hydroxy acids to generate sufficiently strong, red-shifted CD signals for accurate concentration and *er* determination.^30−46^ In particular Schiff base formation with primary amino groups has become a privileged sensing motif, while other functionalities remain challenging.^47−54^ Noteworthy progress has been made with cucurbiturils, pillararenes, calixarenes, and other macrocycles, but they typically generate weak, blue-shifted CD maxima.^55−62^ As a result, methodically new binding and CD induction strategies are needed to extend the current chiroptical sensing space to other classes of compounds.^63^

Quantitative optical sensing of chiral nitriles has been largely unattainable to date. The lack of a small-molecule chiroptical probe that targets nitriles can be attributed to several challenges. Nitriles are weakly coordinating ligands which disfavor stoichiometric binding assays with a chromophoric metal complex. The local *C*_∞*v*_ symmetry of the linear cyano group and the free rotation around its axis impede well-defined stereochemical interactions and distinct CD induction upon binding to a sensor. In addition, the considerable distance between the stereogenic center and the metal-coordinating nitrogen atom further diminishes effective chirality imprinting onto the metal complex, which therefore remains unlikely to generate a strong chiroptical response to the binding event. We now show how these difficulties can be overcome with a novel reaction-based sensing assay in which the commercially available Schwartz reagent plays a critical role to desymmetrize the nitrile group into a rigid iminate that is readily transmetalated from the zirconium center to a chromophoric palladium complex. This process induces a strong, red-shifted chiroptical signal that is directly correlated to the enantiomeric composition of the nitrile substrate. We use an achiral Pd complex as sensor to avoid formation of diastereomers, which simplifies concomitant concentration and *er* analysis, and we demonstrate the viability of this concept with a large variety of chiral nitrile compounds.

At the onset of this study, we followed a previously reported strategy that is based on the self-assembly of CD-active alkene transition metal complexes formed through *in situ* halide abstraction with silver salts in the presence of the sensing target, which proved highly successful for chiroptical terpene and terpenoid analysis (Figure 1).^64^ Comprehensive screening of 25 sensor candidates comprising a series of metal halides and dihalides using silver tetrafluoroborate to generate a vacant binding site and (*S*)-2-methylbutyronitrile as a test analyte showed first hits and induced CD (ICD) signals in chlorinated solvents (SI). However, this protocol failed when (*S*)-2-(naphthalene-2-yl)propanenitrile was employed, indicating a limited application scope. We hypothesized that the major challenge was not to form metal coordination complexes with the nitrile compounds but that the rotational freedom and the local *C*_∞*v*_ symmetry of the linear cyano group would considerably diminish the chirality imprinting onto the chromophoric metal sensor, which was considered a crucial prerequisite for strong CD inductions. We therefore decided to address these issues with a methodologically different approach and investigated the possibility of chiroptical nitrile sensing via hydrozirconation and subsequent transmetalation of the iminate moiety, which would exhibit a desymmetrized structure with less rotational freedom, to a chromophoric metal halide complex. Indeed, this appeared to work under anhydrous conditions with several metal complexes and particularly well with [(η^3^-1-*tert*-butylindenyl)(μ-Cl)Pd]_2_, **3** (Figure 2).

**Figure 1:**
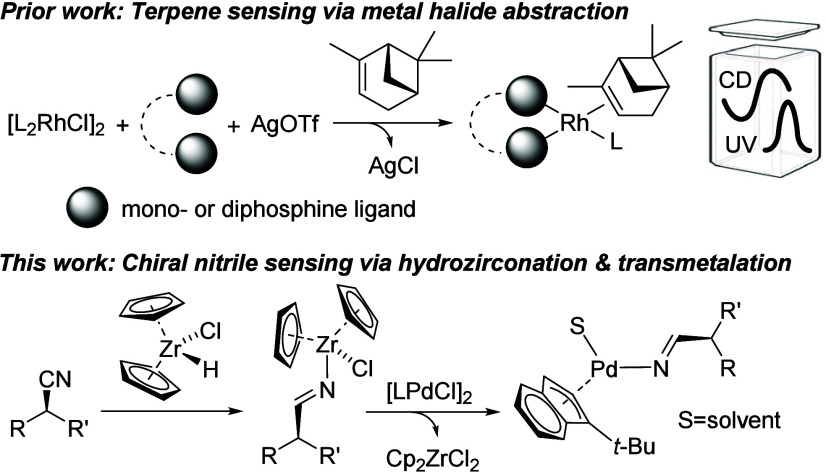
Chiroptical sensing strategies for weakly nucleophilic compounds.

**Figure 2:**
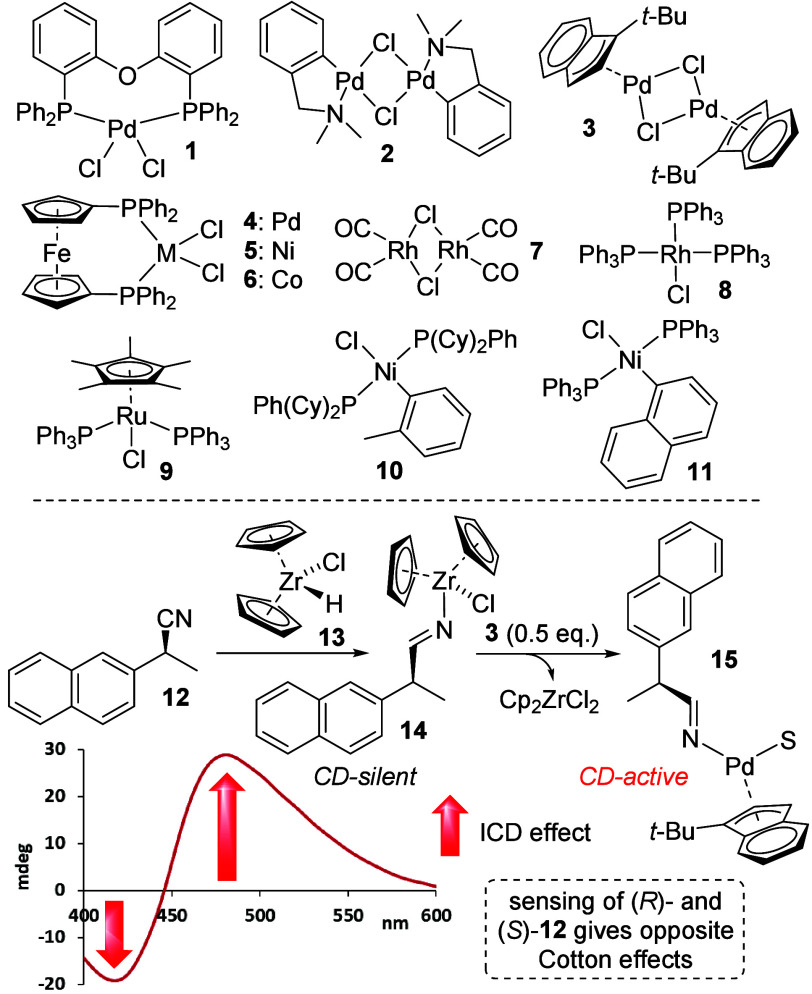
Representative examples of sensors screened (top). Chiroptical nitrile sensing via hydrozirconation and transmetalation to palladium complex **3** (bottom). The CD spectrum was obtained at 0.8 mM in CH_2_Cl_2_. S = solvent.

We were pleased to observe that the reaction between nitrile **12** and Schwartz reagent **13** followed by transmetalation to the Pd complex **3** yields red-shifted ICD maxima beyond 400 nm, which is advantageous because it reduces the risk of possible interferences when chiral impurities that typically display chiroptical effects at shorter wavelengths are present and it simplifies the adaption to automated multiwell plate readers that are known to allow high-throughput screening of hundreds of samples but have technical problems with recording CD signals in the region around 400 nm.^65^

Having developed the first chiral nitrile sensing method, we decided to investigate the mechanistic features of the chemical-reaction-based assay and the underlying chirality recognition. As mentioned above, CD sensing of chiral alkenes devoid of any other functional group via metal coordination, for example by *in situ* halide abstraction of [(Ph_3_P)_3_Rh(I)Cl], **8**, is known to give strong CD effects, but this strategy failed when applied to nitrile compounds. We were able to show that stoichiometric metal coordination indeed occurs under similar conditions by growing a single crystal derived from (*S*)-2-methylbutyronitrile, **16**, which turned out to be CD-silent (Figure 3). A closer look at the crystal structure reveals that the end-on nitrile binding motif places the chirality center remote from the propeller-like triphenylphosphine Rh ligands. This supports our initial hypothesis that metal coordination occurs and that the lack of CD induction is likely a result of insufficient chirality imprinting on the sensor. We then turned our attention to the hydrozirconation reaction. NMR monitoring showed that this is a fast process, and the addition of Cp_2_ZrHCl to 2-phenylpropanenitrile, **18**, was quantitative and complete within 5 min without byproduct formation. This reaction is characterized by an upfield shift of the methyl and methine protons in the reduced nitrile substrate and by the appearance of the characteristic imine proton at around 8.5 ppm. We suspected that formation of rapidly interconverting *E*/*Z*-zirconium iminate isomers is possible, and this was confirmed by variable-temperature NMR experiments (SI). Unfortunately, attempts to follow the transmetalation step NMR spectroscopically gave inconclusive results. But we were able to grow a single crystal of Cp_2_ZrCl_2_ directly from the reaction mixture, which corroborates the proposed reaction pathway (SI). UV/CD and ESI-MS experiments verified that the iminate formation is essential for the optical nitrile sensing and that it is transferred to the indenylpalladium complex, which may also carry a solvent molecule.

**Figure 3:**
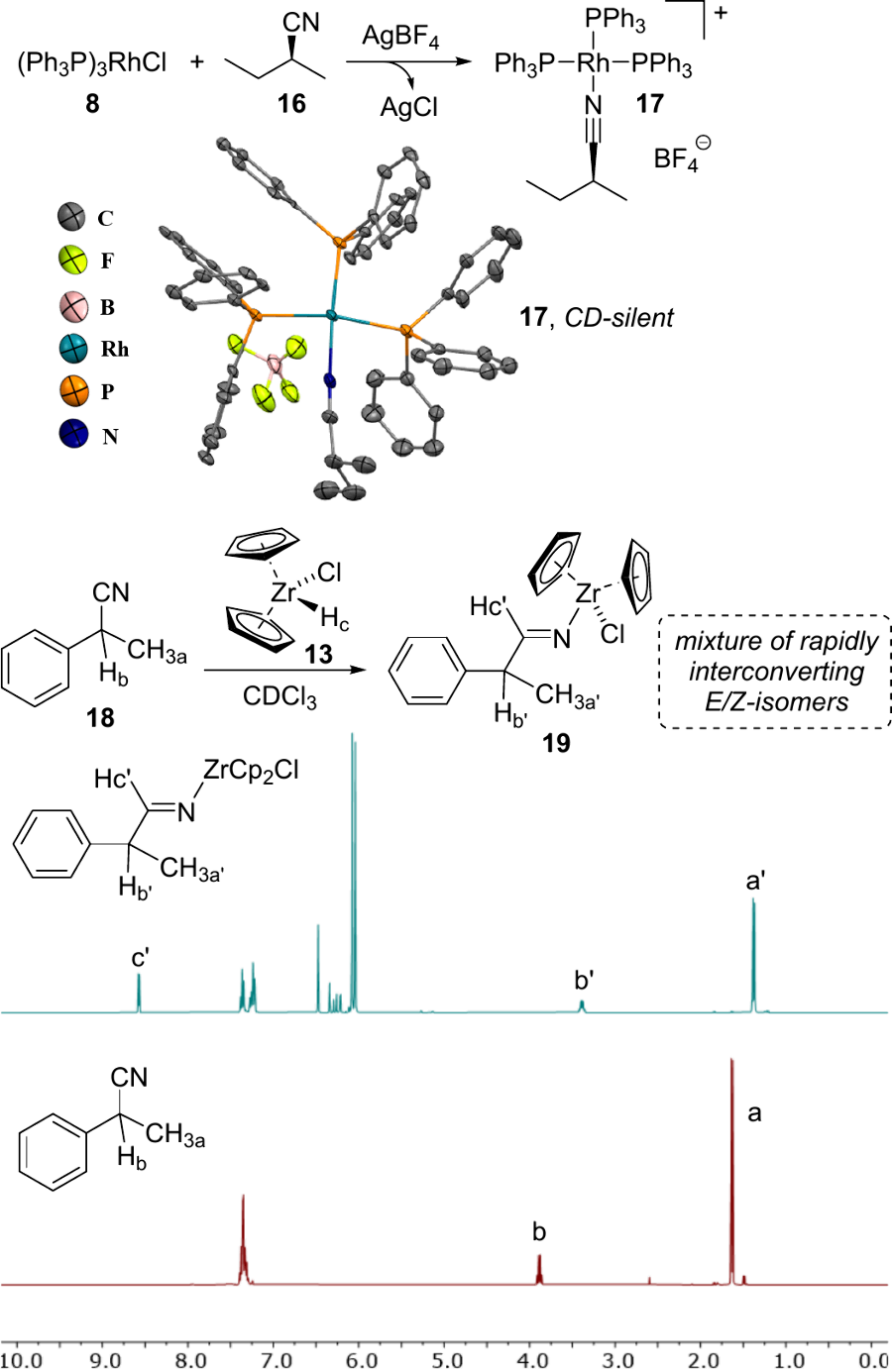
Halide abstraction and coordination of (*S*)-2-methylbutyronitrile using (PPh_3_)_3_RhCl (top) and an NMR study of the hydrozirconation of 2-phenylpropanenitrile (bottom). The hydrogens are omitted in the crystal structure for clarity. See SI for details.

Additional ICD time and stoichiometry experiments showed that the formation of a Pd complex exhibiting equimolar amounts of the indenyl ligand and the iminate is complete after 9 h. We note that these findings are in agreement with the monomeric palladium complex **15**, while the formation of a dinuclear complex cannot be excluded. Importantly, the rhodium complex **8**, which failed to give an ICD effect in the halide abstraction procedure, gave a distinct chiroptical response, albeit not as strong as **3**, when it was applied in the hydrozirconation/transmetalation method.

The chiral nitriles shown in Figure 4 were used to evaluate the scope of our chiroptical sensing method. These compounds comprise purely aliphatic scaffolds, structures with various aromatic rings that were prepared according to a literature protocol^66^ as well as multifunctional substrates and pharmaceutically relevant ones. Compound **26** is a precursor to isavuconazole, an antifungal drug, and pregnenolone 16α-carbonitrile, **27**, is a steroidal antiglucocorticoid and a pregnane receptor agonist. In all cases, red-shifted ICD signals were measured which demonstrates the broad utility and functional group tolerance of ketone, alcohol, ester, carbamate, alkene, and heterocyclic structures (SI).

**Figure 4:**
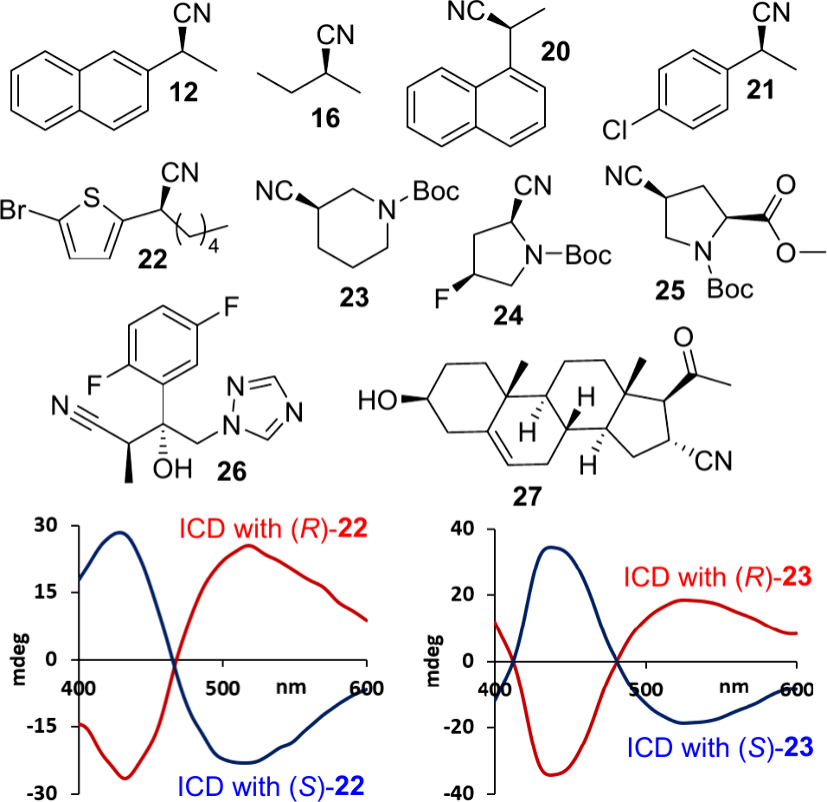
Structures of chiral nitrile compounds used in this study and ICD effects obtained by sensing the enantiomers of **22** and **23**. Only one enantiomer is shown. See SI for details.

Finally, 10 samples containing **12** at varying concentrations and enantiomeric compositions were prepared to test the use of the chiroptical nitrile sensing protocol (SI). To achieve this, we analyzed CD induction effects and concomitant UV changes. The ICD maxima generated by the hydrozirconation–transmetalation sequence can be directly correlated to the enantiomeric ratio of the nitrile analyte with the help of a calibration curve. Because we are using an achiral sensor, we avoid formation of new diastereoisomers during the Pd coordination. This greatly simplifies the analytical task and eliminates complications regarding potentially erroneous *dr* to *er* conversions, and we can take advantage of the inherently enantioselective nature of CD spectroscopy to determine enantiomeric ratios, [*R*]/[*S*].

Simultaneous changes observed in the UV spectra, however, are nonenantioselective, i.e., independent of the enantiomeric sample composition, and therefore allow determination of the total analyte concentration, [*R*]+[*S*]. The results of this comprehensive CD/UV sensing concept are listed in Table 1. In general, the nitrile analysis gives accurate concentration and *er* values with error margins that would not allow analysis of near-racemic samples but are comparable to previously reported optical sensing methods.^29^

**Table 1:** Quantitative Sensing of the Concentration and Enantiomeric Ratio of 10 Samples of Nitrile **12**

	Actual Composition		CD Sensing Results	
Sample #	Conc (mM)	*er* (*R:S*)	Conc (mM)	*er* (*S:R*)
1	22.50	93.5:6.5	20.80	97.0:3.0
2	12.50	10.0:90.0	15.60	13.5:86.5
3	23.75	65.0:35.0	24.50	61.0:39.0
4	10.00	97.5:2.5	8.00	98.0:2.0
5	17.50	70.0:30.0	18.80	66.5:33.4
6	15.00	82.5:17.5	14.60	82.0:17.0
7	20.00	21.0:79.0	19.40	21.5:78.5
8	8.00	0.0:100.0	8.40	1.0:99.0
9	18.00	85.0:15.0	18.90	79.0:21.0
10	21.00	12.5:87.5	20.10	8.0:92.0

In summary, we have demonstrated that the chiroptical sensing of nitriles is possible via hydrozirconation and subsequent transmetalation of the corresponding iminate to a chromophoric palladium complex. This strategy overcomes previously unaddressed challenges with nitrile sensing, e.g., the local *C*_∞*v*_ symmetry of the linear cyano group and the free rotation about its axis, that weaken chirality imprinting onto metal coordination complexes, a widely accepted prerequisite for strong chiroptical signal induction. Using Schwartz reagent and [(η^3^-1-*tert*-butylindenyl)(μ-Cl)Pd]_2_, which are both commercially available, a continuous workflow that yields red-shifted circular dichroism inductions and characteristic UV changes with a variety of substrates including multifunctional scaffolds and pharmaceutically relevant molecules was introduced. The utility of this protocol was highlighted with the determination of the enantiomeric composition and total concentration of 10 chiral nitrile samples. The optical assay is compatible with generally available high-throughput experimentation equipment and multiwell CD plate readers if parallel analysis of hundreds of samples is desirable.

## Data Availability

The data underlying this study are available in the published article and its Supporting Information.

## Supporting Information Available

Experimental details, product characterization, CD, UV, X-ray, and NMR spectra (PDF)
