# Supplementary material for: Optical Enantiodifferentiation of Chiral Nitriles
Source: Org Lett. 2024 Sep 4;26(36):7644–9. doi: 10.1021/acs.orglett.4c02758 (PMC11406584; doi:10.1021/acs.orglett.4c02758)
Supplement: Supplementary file 1 — ol4c02758_si_001.pdf [file ol4c02758_si_001.pdf]

Supporting Information

Optical Enantiodifferentiation of Chiral Nitriles

Jeffrey S. S. K. Formen and Christian Wolf*
Department of Chemistry, Georgetown University, 37th and O St., Washington DC 20057, USA.
Email: cw27@georgetown.edu

Contents	Page
1. General information	S2
2. Sensor development	S3
2.1. Chiroptical nitrile sensing via halide abstraction	S3
2.2. Chiroptical nitrile sensing via *in-situ* self-assembly	S6
2.3. Chiroptical nitrile sensing via hydrozirconation and transmetalation	S7
3. Mechanistic investigations	S12
3.1. Hydrozirconation study	S12
3.2. Transmetalation reaction analysis	S14
3.3. Mass spectrometry	S20
4. Nitrile substrate scope	S21
5. Quantitative nitrile sensing: absolute configuration, enantiomeric excess, and total concentration	S29
6. Asymmetric nitrile synthesis	S32
7. References	S46

1. General information

All commercially available reagents and solvents were used without further purification. Reactions were carried out under inert and anhydrous conditions. Flash chromatography was performed on silica gel, particle size 40-63 mm. NMR spectra were obtained on a Varian instrument at 400 MHz (^{1}H NMR) and 100 MHz (^{13}C NMR) at room temperature using $CDCl_3$ as solvent. Chemical shifts are reported in ppm relative to the solvent peaks. CD spectra were collected with a standard sensitivity of 100 mdeg, a data pitch of 1.0 nm, and a bandwidth of 1.0 nm in a continuous scanning mode with a scanning speed of 200 nm/min and a response of 1.0 s (1 cm path length). The data were baseline corrected and smoothed using a binomial equation. Chiral GC-MS measurements were acquired on an Agilent 5977C GC/MSD equipped with a 2,6-dimethyl-3-pentyl-beta-cyclodextrin (50% in polysiloxane) coated capillary (30 m, 0.25 mm, 0.125 μm). ESI/MS analysis was performed by direct injection into a single quadrupole LC/MSD iQ. Single crystal analysis was performed at 100 K using a Siemens platform diffractometer with a graphite monochromated Mo-Ka radiation (λ = 0.71073 Å). Data were integrated and corrected using the APEX 3 program. The structures were solved by direct methods and refined with full-matrix least-square analysis using SHELXL-2019/1 software. HR-MS data were obtained using electron spray ionization time-of-flight (ESI-TOF) spectrometry.

2. Sensor development

2.1. Chiroptical nitrile sensing via halide abstraction

Metal dihalide (L_2MX_2) complexes

To a solution of (*S*)-2-methylbutyronitrile (4.2 mg, 0.05 mmol) and sensors **1-7** and **28-36** (0.025 mmol) in 1.0 mL CH_2Cl_2 was added $AgBF_4$ (9.9 mg, 0.05 mmol) as a solid. The mixture was stirred for 1 hour and subsequently filtered through a 2-micron syringe filter. The resulting clear solution was then subjected to CD analysis. No CD signals were observed. The same protocol was repeated with 0.05 mmol of each sensor. Again, no CD inductions were observed.

Scheme S1. Chiroptical sensing of (*S*)-2-methylbutyronitrile with sensors **1-7** and **28-36**.

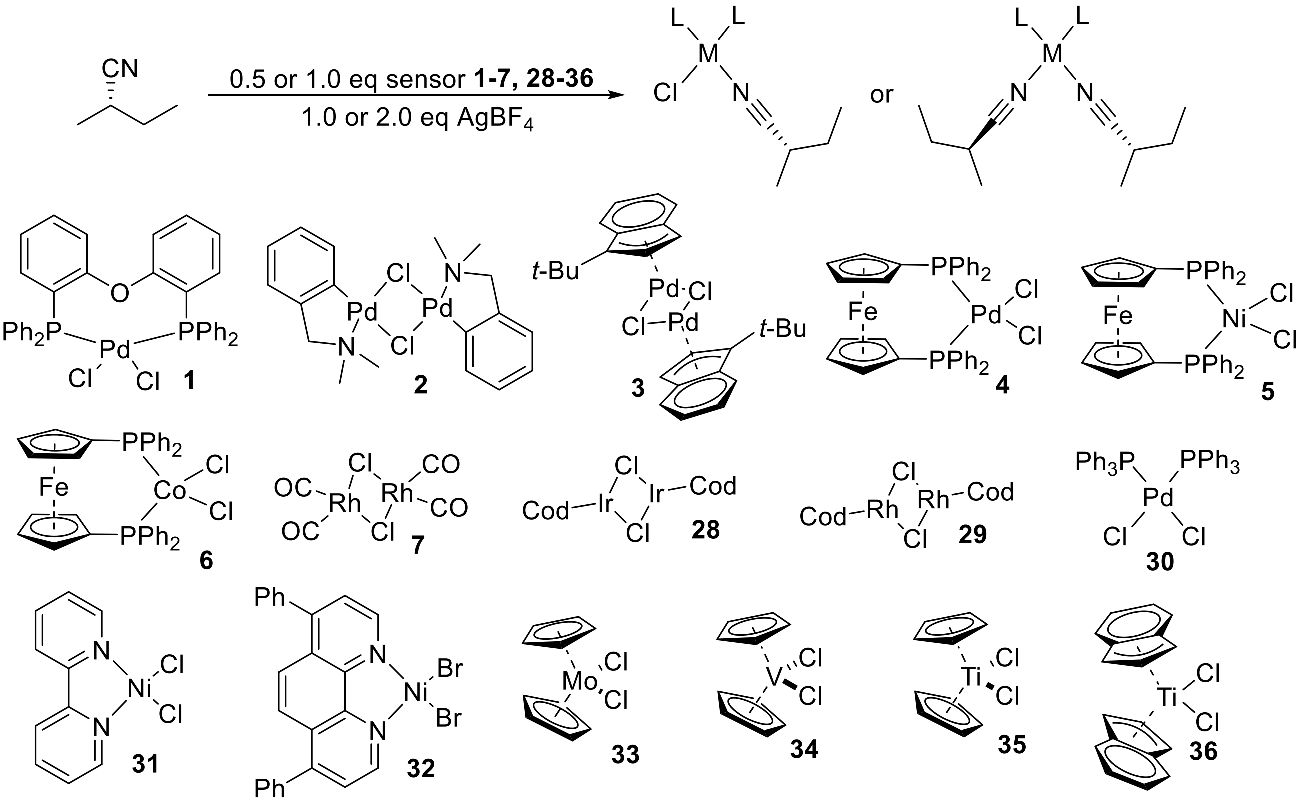

Metal monohalide ($L_{3-5}MX$) complexes

To a solution of (*S*)-2-methylbutyronitrile (4.2mg, 0.05mmol) and sensors **8-11** and **37-41** (0.05 mmol) in 1.0 mL CH_2Cl_2 was added $AgBF_4$ (9.9 mg, 0.05 mmol) as a solid. The mixture was stirred for 1 hour and subsequently filtered through a 2-micron syringe filter. The resulting clear

solution was then subjected to CD analysis. Weak, red-shifted CD signals were observed with sensors **20-22**.

Scheme S2. Chiroptical sensing of (*S*)-2-methylbutyronitrile with sensors **8-11**, **37-41**.

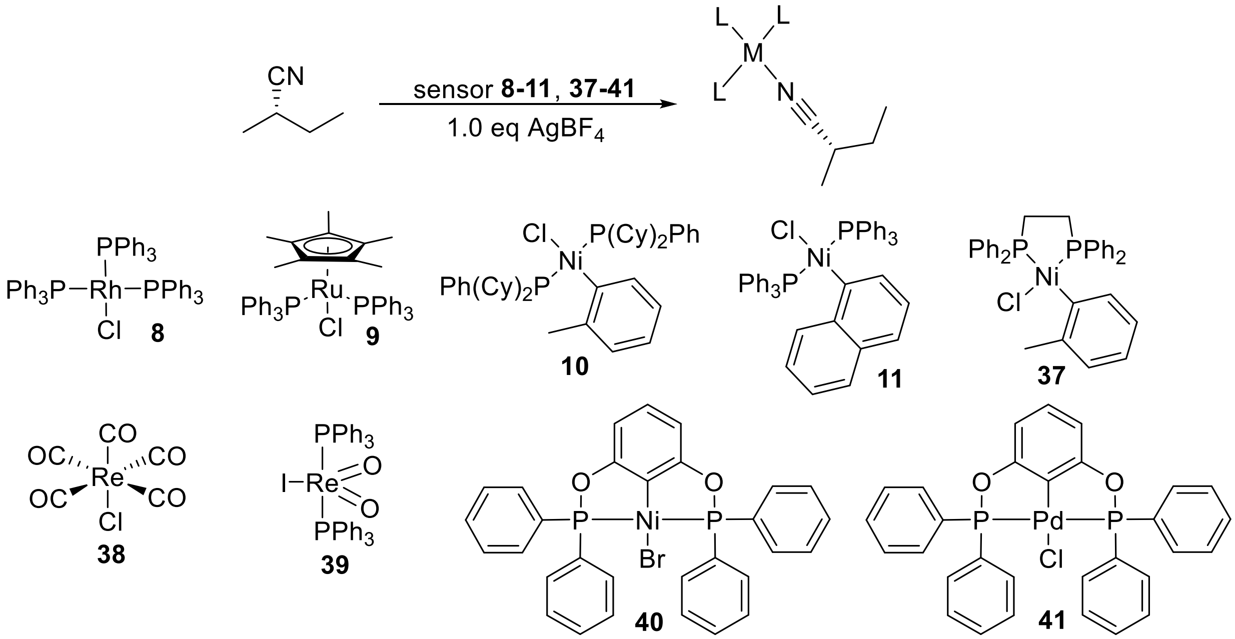

Figure S1. Chiroptical sensing of (*S*)-2-methylbutyronitrile with sensor **8**.

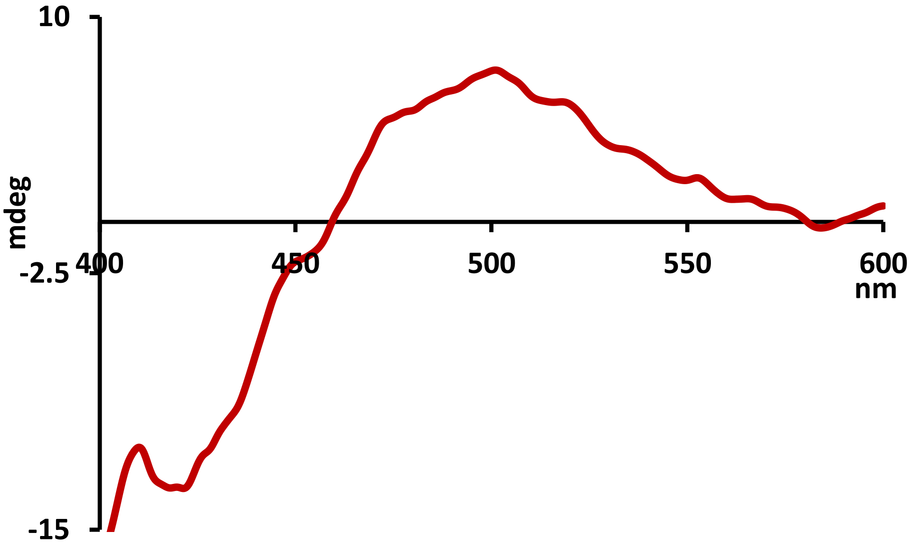

CD measurements were taken at 3.0 mM in CH_2Cl_2.

Figure S2. Chiroptical sensing of (*S*)-2-methylbutyronitrile with sensor **10**.

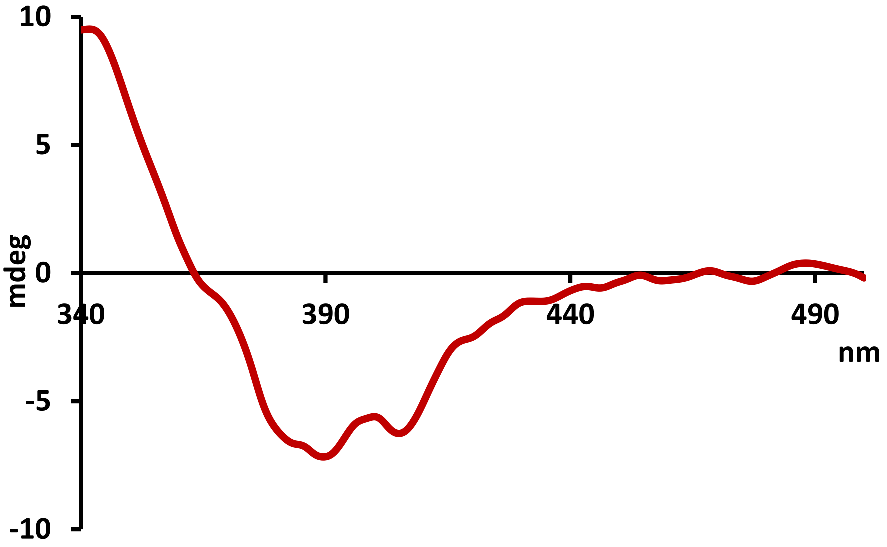

CD measurements were taken at 2.5 mM in CH_2Cl_2.

.

Figure S3. Chiroptical sensing of (*S*)-2-methylbutyronitrile with sensor **37**.

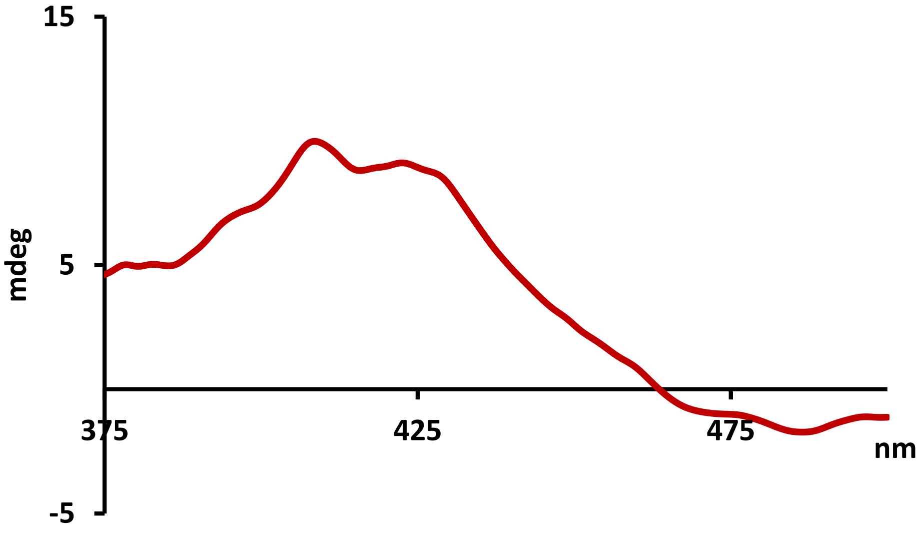

CD measurements were taken at 2.5 mM in CH_2Cl_2.

Scheme S3. Chiroptical sensing of (*S*)-2-(naphthalene-2-yl)propanenitrile with sensors **9**, **10**, and **37**.

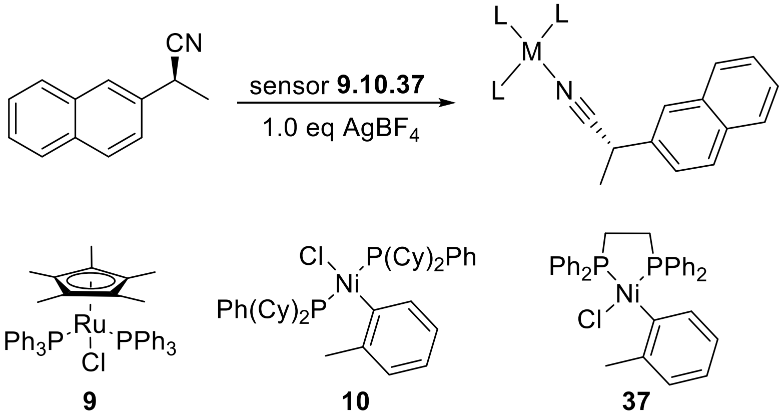

To a solution of (*S*)-2-(naphthalene-2-yl)propanenitrile (9.1mg, 0.05mmol) and sensors **9, 10, 37** (0.05 mmol) in 1.0 mL CH_2Cl_2 was added $AgBF_4$ (9.9 mg, 0.05 mmol) as a solid. The mixture was stirred for 1 hour and subsequently filtered through a 2-micron syringe filter. The resulting clear solution was then subjected to CD analysis. No CD signals were observed.

2.2. Chiroptical nitrile sensing via *in-situ* self-assembly

A stock solution of PPh_3 (20.0 mM in CH_2Cl_2) was prepared and 0.5 mL portions were placed in 8.0 mL vials. In separate vials, a stock solution of $[Rh(cod)Cl]_2$ (12.5 mM in CH_2Cl_2), $[Ir(cod)Cl]_2$ (12.5 mM in CH_2Cl_2) and (*S*)-2-methylbutyronitrile (20.0 mM in CH_2Cl_2) were prepared. To each vial containing 0.5 mL of the phosphine ligand were added 0.5 eq (200.0 μL) of the Rh(I) or Ir(I) dimer complex followed by 2.0 eq of $AgBF_4$ (4.0 mg, 0.02 mmol). The mixtures were stirred for 1 hour and subsequently filtered through a 2-micron syringe filter. Binding of the analyte was confirmed by X-ray crystallography, but no CD signal was observed for either species.

Scheme S4. Chiroptical sensing of (*S*)-2-methylbutyronitrile via *in-situ* self-assembly of Rh and Ir complexes

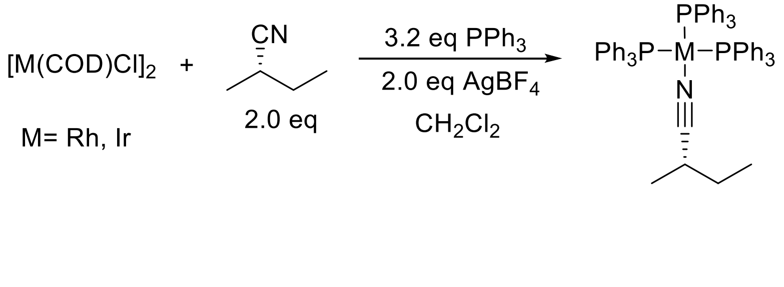

2.3. Chiroptical nitrile sensing via hydrozirconation and transmetalation

Scheme S5. Chiroptical sensing of (*S*)-2-(naphthalene-2-yl)propanenitrile via hydrozirconation and transmetalation with sensors **1-11** and **28-41**.

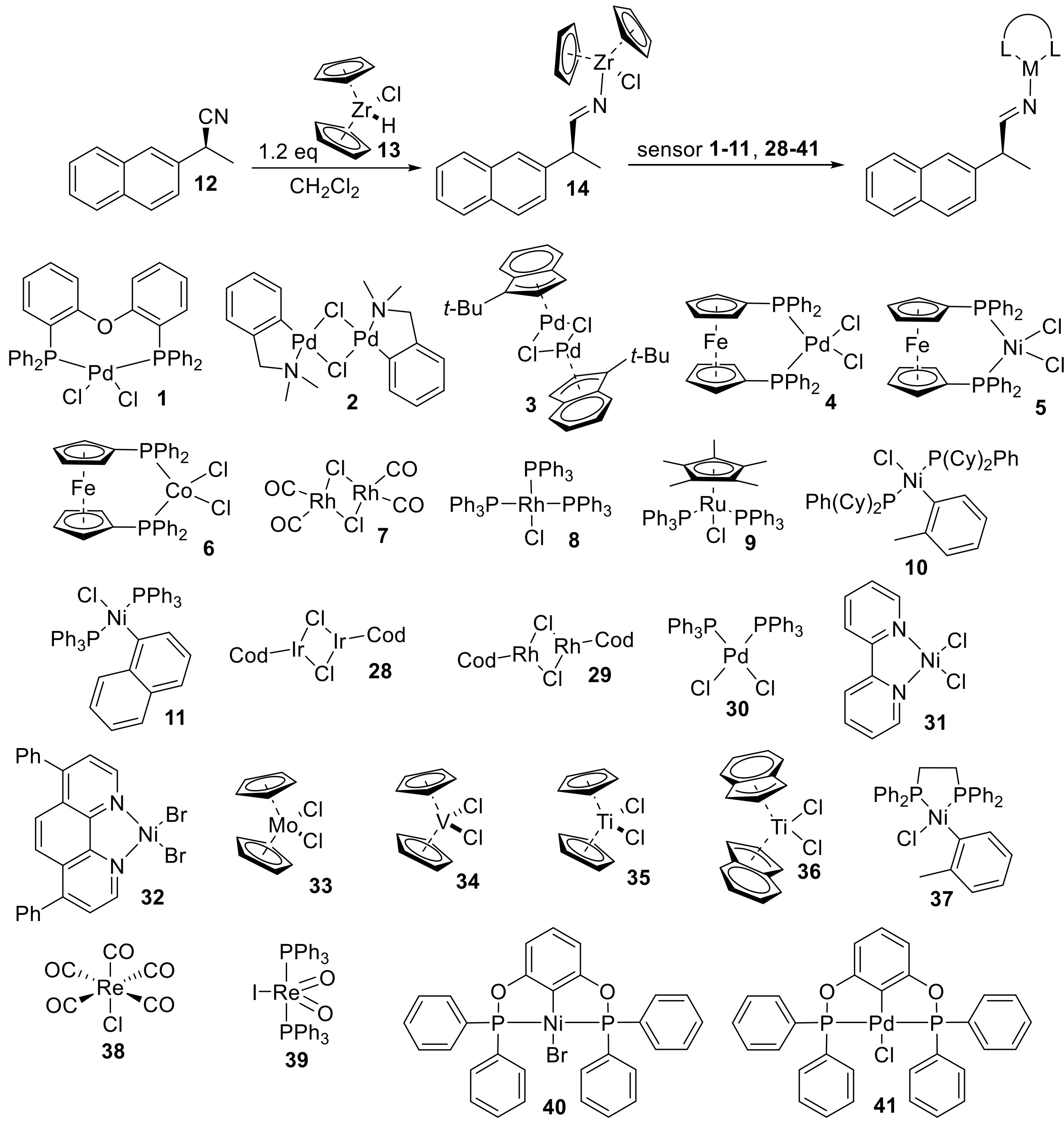

To a solution of (*S*)-2-(naphthalene-2-yl)propanenitrile (3.6mg, 0.02 mmol) in 1.0 mL CH_2Cl_2 was added zirconocene chloride hydride (6.4 mg, 0.022 mmol) as a solid. After 5 minutes of stirring, the suspension turned into a yellow solution, however no CD signal was observed. Sensors **1-7** and **28-36** were then added in 0.5 eq. and sensors **8-11** and **37-41** were added in 1.0 eq. as solids. The solution was allowed to stir overnight and was subjected to CD analysis at the concentration indicated underneath each spectrum. Sensor **3** gave the strongest CD signal with a maximum of 29 mdeg at 480 nm at 0.8 mM in CH_2Cl_2. Thus, further optimization were carried out with **3**.

Figure S4. Chiroptical sensing of (*S*)-2-(naphthalene-2-yl)propanenitrile via hydrozirconation and transmetalation with sensor **2**.

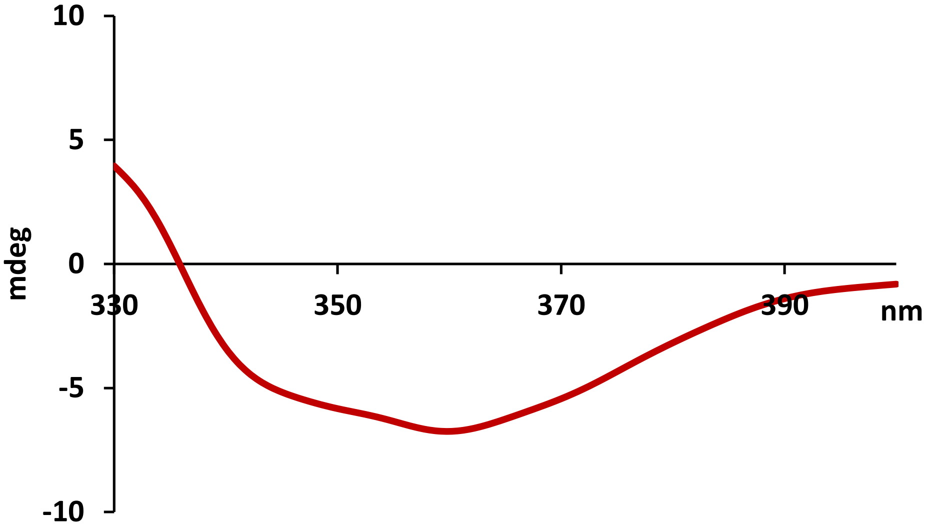

CD measurements were taken at 0.9 mM in CH_2Cl_2.

Figure S5. Chiroptical sensing of (*S*)-2-(naphthalene-2-yl)propanenitrile via hydrozirconation and transmetalation with sensor **3**.

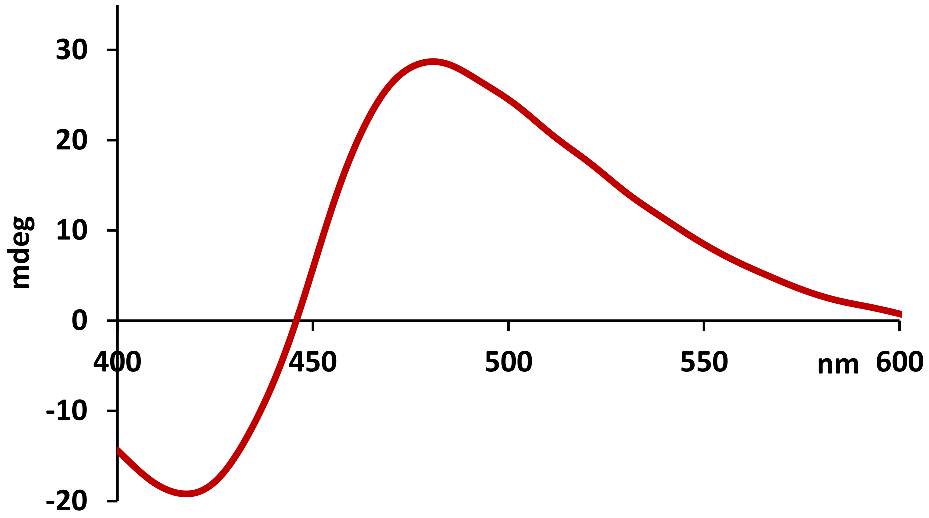

CD measurements were taken at 0.8 mM in CH_2Cl_2.

Figure S6. Chiroptical sensing of (*S*)-2-(naphthalene-2-yl)propanenitrile via hydrozirconation and transmetalation with sensor **28**.

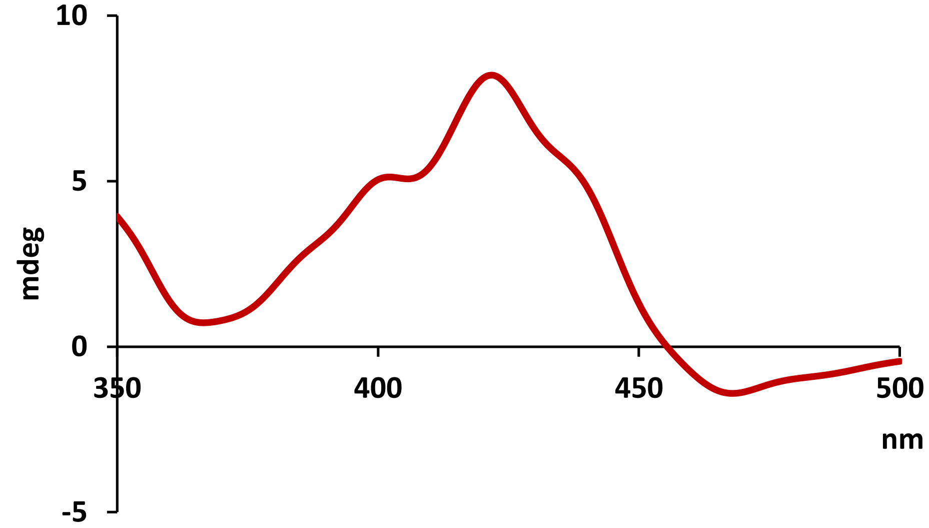

CD measurements were taken at 1.9 mM in CH_2Cl_2.

Figure S7. Chiroptical sensing of (*S*)-2-(naphthalene-2-yl)propanenitrile via hydrozirconation and transmetalation with sensor **7**.

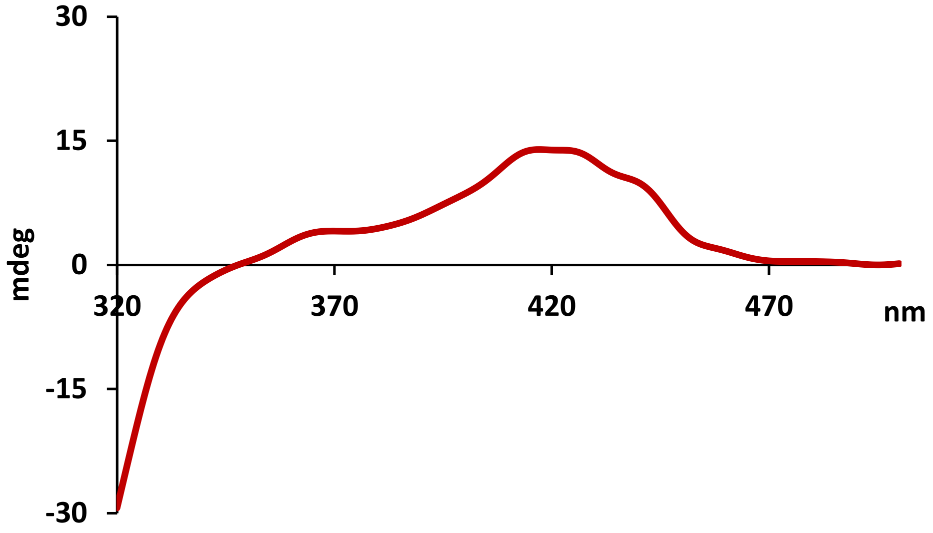

CD measurements were taken at 0.6 mM in CH_2Cl_2.

Figure S8. Chiroptical sensing of (*S*)-2-(naphthalene-2-yl)propanenitrile via hydrozirconation and transmetalation with sensor **8**.

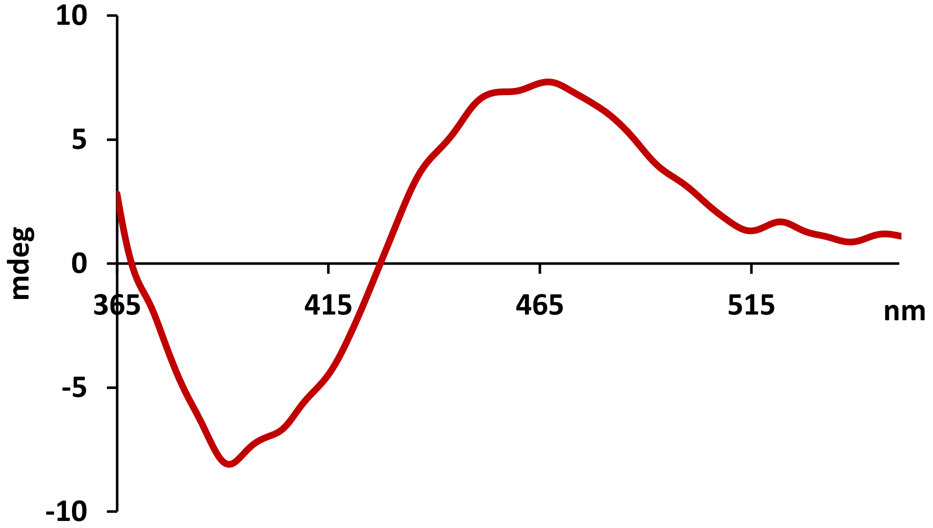

CD measurements were taken at 0.6 mM in CH_2Cl_2.

Figure S9. Chiroptical sensing of (*S*)-2-(naphthalene-2-yl)propanenitrile via hydrozirconation and transmetalation with sensor **10**.

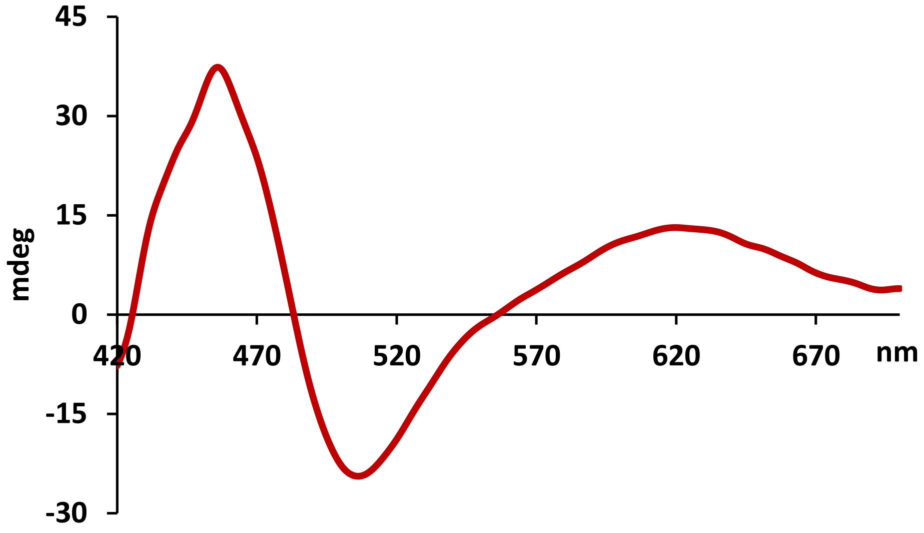

CD measurements were taken at 2.5 mM in CH_2Cl_2.

Figure S10. Chiroptical sensing of (*S*)-2-(naphthalene-2-yl)propanenitrile via hydrozirconation and transmetalation with sensor **11**.

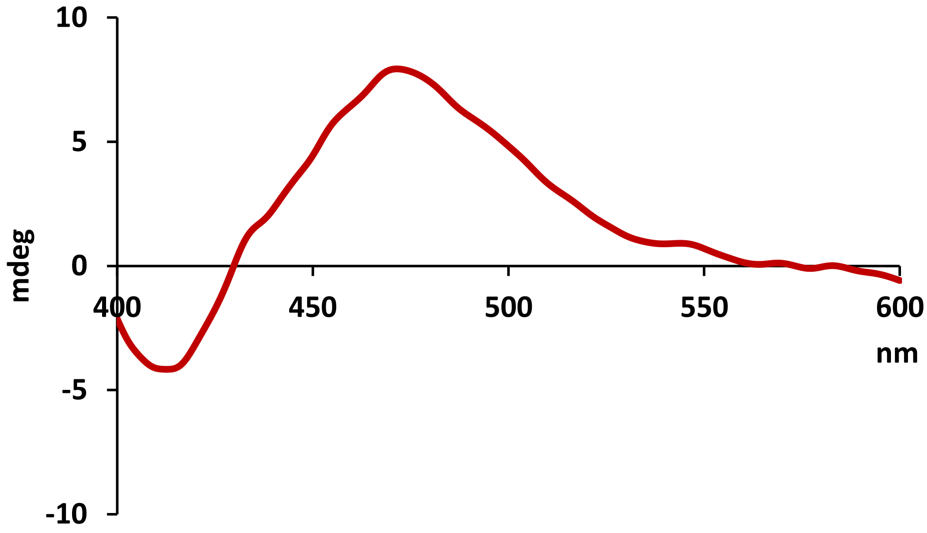

CD measurements were taken at 3.4 mM in CH_2Cl_2.

3. Mechanistic investigations

3.1. Hydrozirconation study

To a solution of 2-phenylpropanenitrile (10.0 mg, 0.08 mmol) in 1.0 mL $CDCl_3$ was added zirconocene chloride hydride (24.5 mg, 0.08 mmol) as a solid. Complete hydrozirconation was observed after 5 minutes.

Scheme S6. ^{1}H NMR analysis of the reaction between 2-phenylpropanenitrile and **13**.

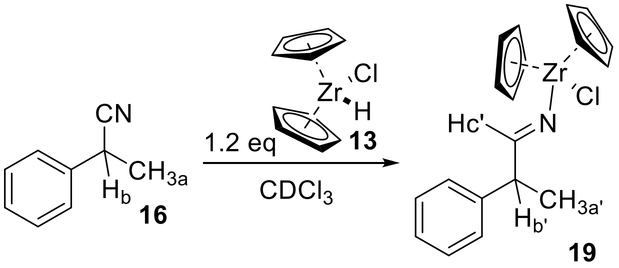

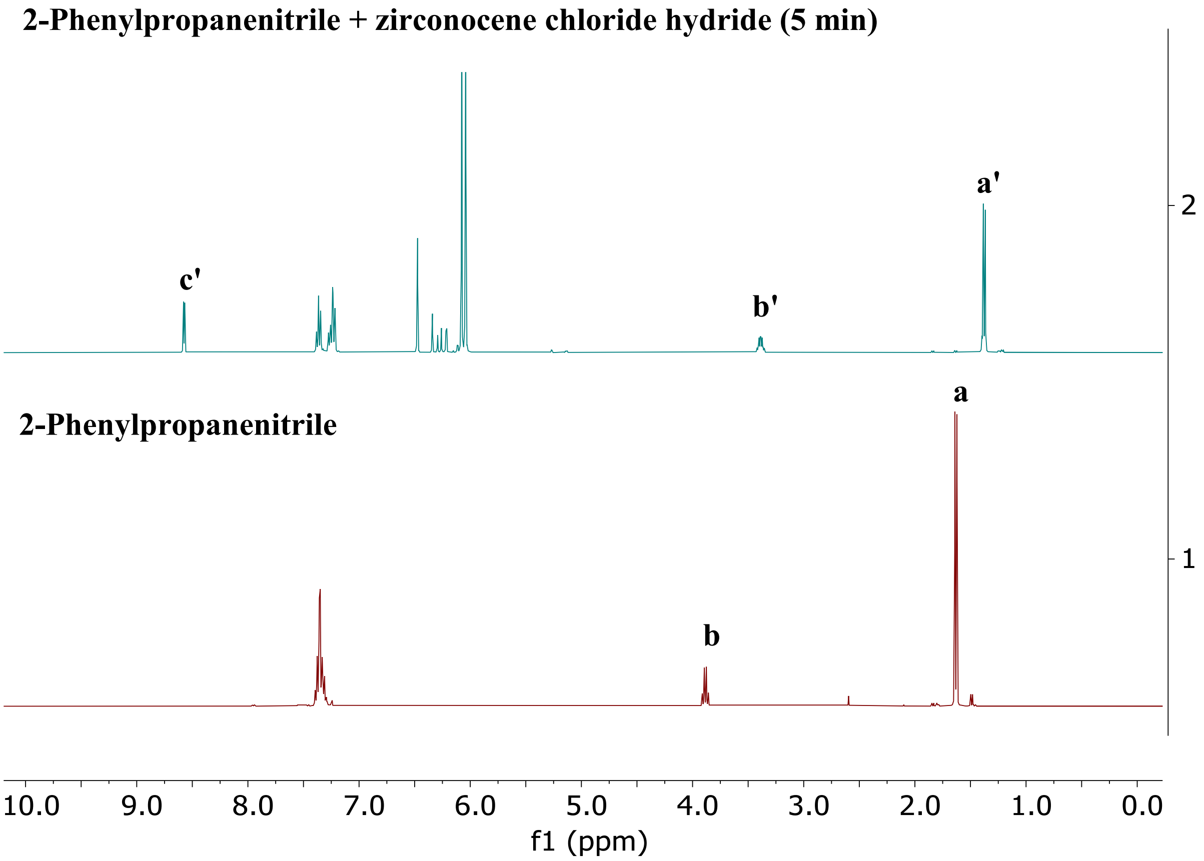

To a solution of 2-phenylpropanenitrile (10.0 mg, 0.08 mmol) in 1.0 mL $CDCl_3$ was added zirconocene chloride hydride (24.5 mg, 0.08 mmol) as a solid. Complete hydrozirconation was observed after 5 minutes. Variable temperature ^1H NMR studies were conducted between 25 °C and -70 °C. At -40 °C partial signal resolution of the rapidly interconverting ***E*-19** and ***Z*-19** isomers was observed. At -60 °C full resolution of ***E*-19** and ***Z*-19** was obtained.

Scheme S7. NMR analysis of the rapid isomerization of ***E*-28** and ***Z*-28**.

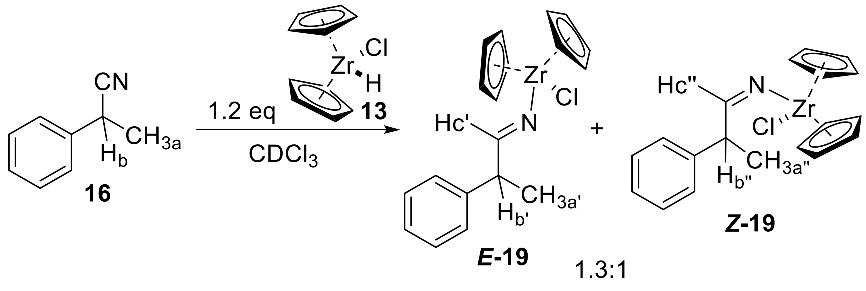

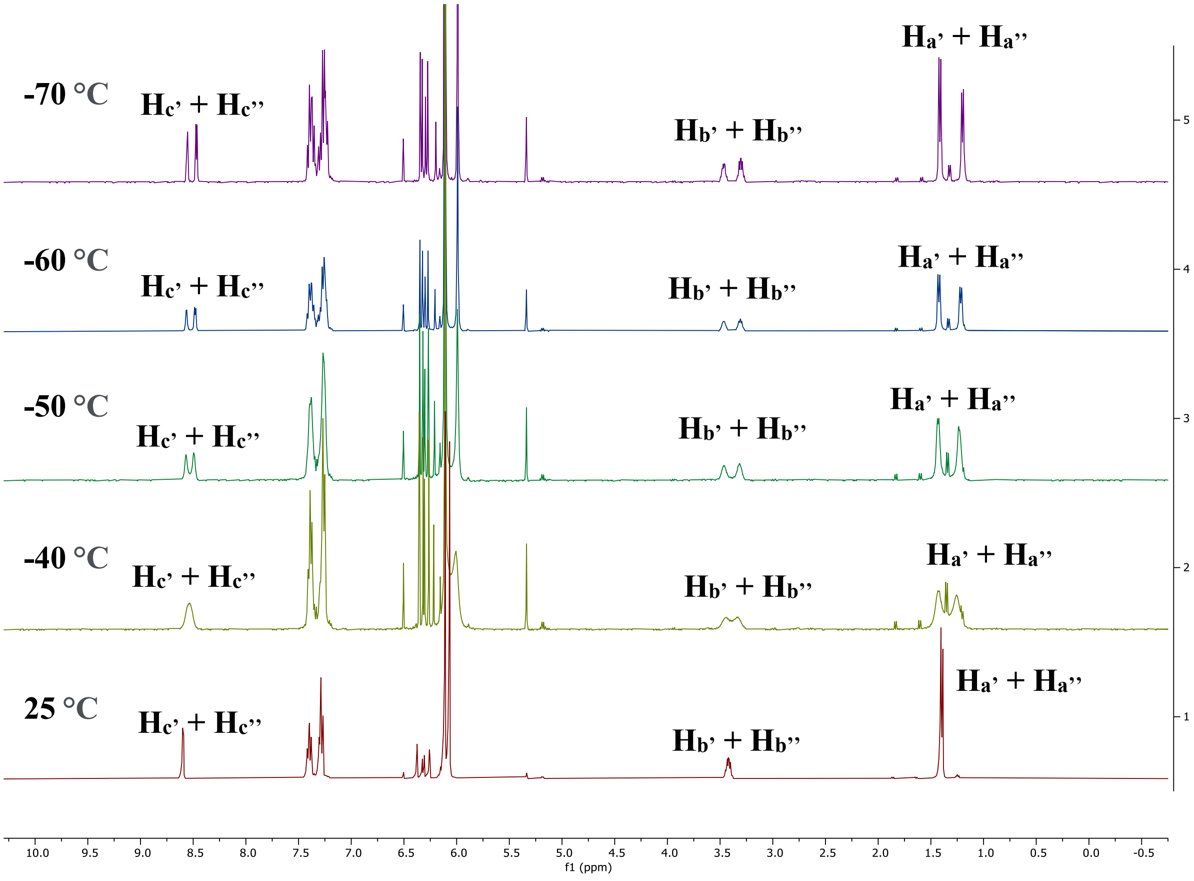

3.2. Transmetalation reaction analysis

To a solution of (*S*)-1-(naphthalen-1-yl)propanenitrile (5.0, 0.03 mmol) in 1.0 mL of CH_2Cl_2 was added zirconocene chloride hydride (7.1 mg, 0.03 mmol) as a solid. The mixture was stirred for 5 minutes and a 0.5 mL aliquot was transferred into to 2 separate vials. Sensor **3** was added either in 0.5 eq (9.4 mg, 0.015 mmol) or in 1.0 eq (18.8 mg, 0.03 mmol) in 2.5 mL CH_2Cl_2. The mixture was stirred for 18 hours and subjected to CD analysis. No significant difference in the CD intensity was observed.

Figure S11. Effect of sensor equivalence on CD induction.

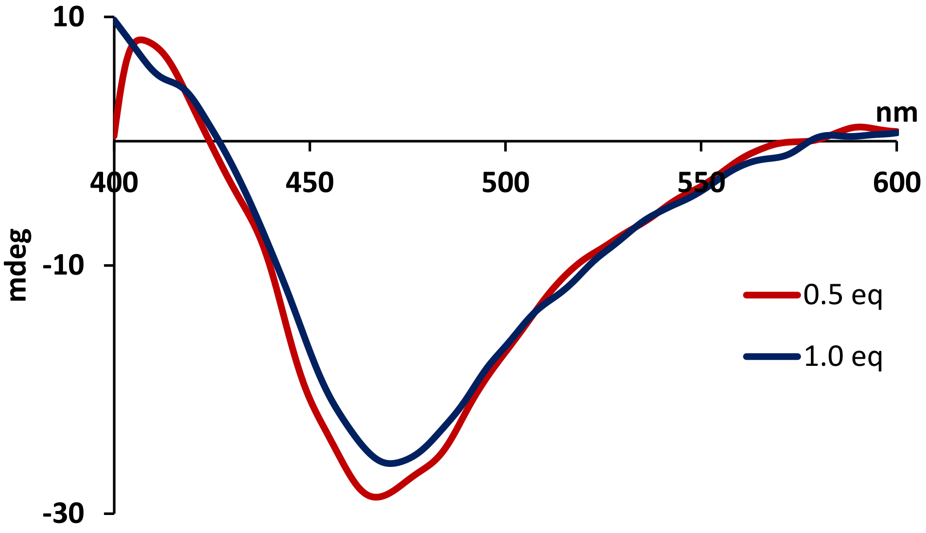

To a solution of 2-phenylpropanenitrile (10.0 mg, 0.08 mmol) in 1.0 mL $CDCl_3$ was added zirconocene chloride hydride (24.5 mg, 0.08 mmol) as a solid. The mixture was stirred for 5 min, after which sensor **3** was added (25.1 mg, 0.04 mmol) in 3.2 mL $CDCl_3$. The mixture was stirred for 18 hours and subjected to ^1H NMR analysis, which was inconclusive.

To a solution of (*S*)-2-(naphthalen-1-yl)propanenitrile (10.0, 0.06 mmol) in 5.0 mL of CH_2Cl_2 was added zirconocene chloride hydride (14.2 mg, 0.06 mmol) as a solid. The mixture was stirred for 5 minutes and a 0.5 mL aliquot was added to 10 separate vials. Sensor **3** was added to each vial (1.7 mg, 0.003 mmol) in 0.5 mL CH_2Cl_2. The samples were subjected to CD analysis in 1 hour increments at 0.75 mM in CH_2Cl_2. The CD intensity did not increase after 9 hours.

Scheme S8. Transmetalation time study

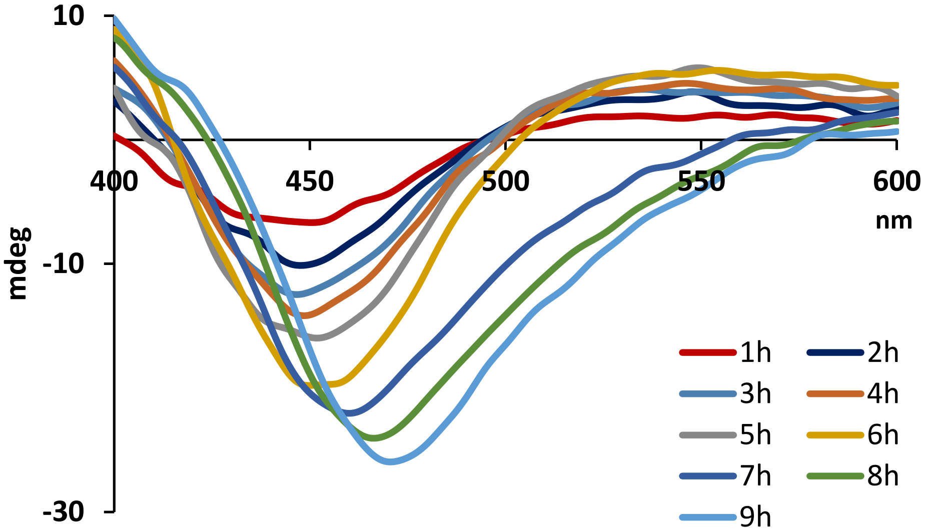

CD measurements were taken at 0.75 mM in CH_2Cl_2.

Cp_2ZrCl_2 transmetalation by-product isolation and analysis

To a solution of 2-phenylpropanenitrile (10.0 mg, 0.08 mmol) in 1.0 mL $CDCl_3$ was added zirconocene chloride hydride (24.5 mg, 0.08 mmol) as a solid. The mixture was stirred for 5 minutes, after which sensor **3** was added (25.1 mg, 0.04 mmol) in 3.2 mL $CDCl_3$. The mixture was stirred for 18 hours and the solvent was removed *in vacuo* under inert atmosphere. The crude product was redissolved in 1.5 mL CH_2Cl_2, and 1.5 mL of pentane was carefully layered on top. White colorless crystals were obtained after 18 hours. Single crystal analysis was performed at 100 K using a Siemens platform diffractometer with a graphite monochromated Mo-Kα radiation (λ = 0.71073 Å). Data were integrated and corrected using the APEX 3 program. The structures were solved by direct methods and refined with full-matrix least-square analysis using SHELXL-2019/1 software. Non-hydrogen atoms were refined with anisotropic displacement parameter. Crystal data: $C_{10}H_{10}Cl_2Zr$, *M*= 292.30, 0.634 x 0.174 x 0.110 mm^3, triclinic, space group P-1, a= 12.9558(14), b= 13.7806 (15), c= 20.363(2) Å, Z = 12.

Scheme S9. X-ray crystallographic analysis of the transmetalation byproduct.

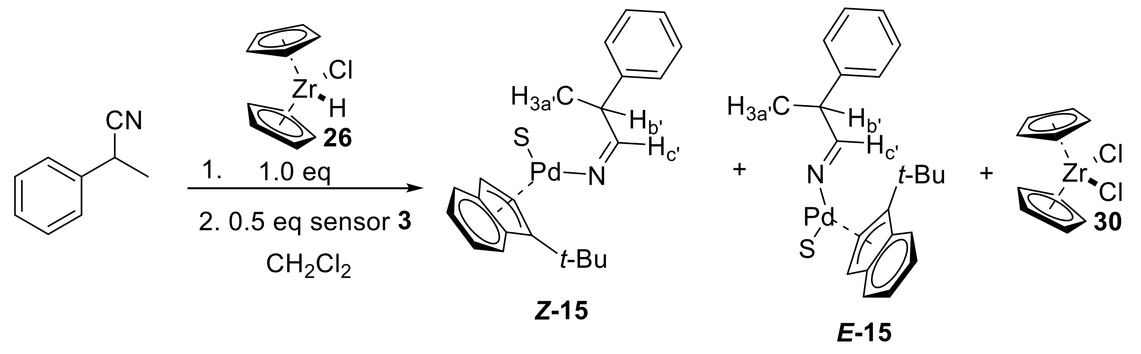

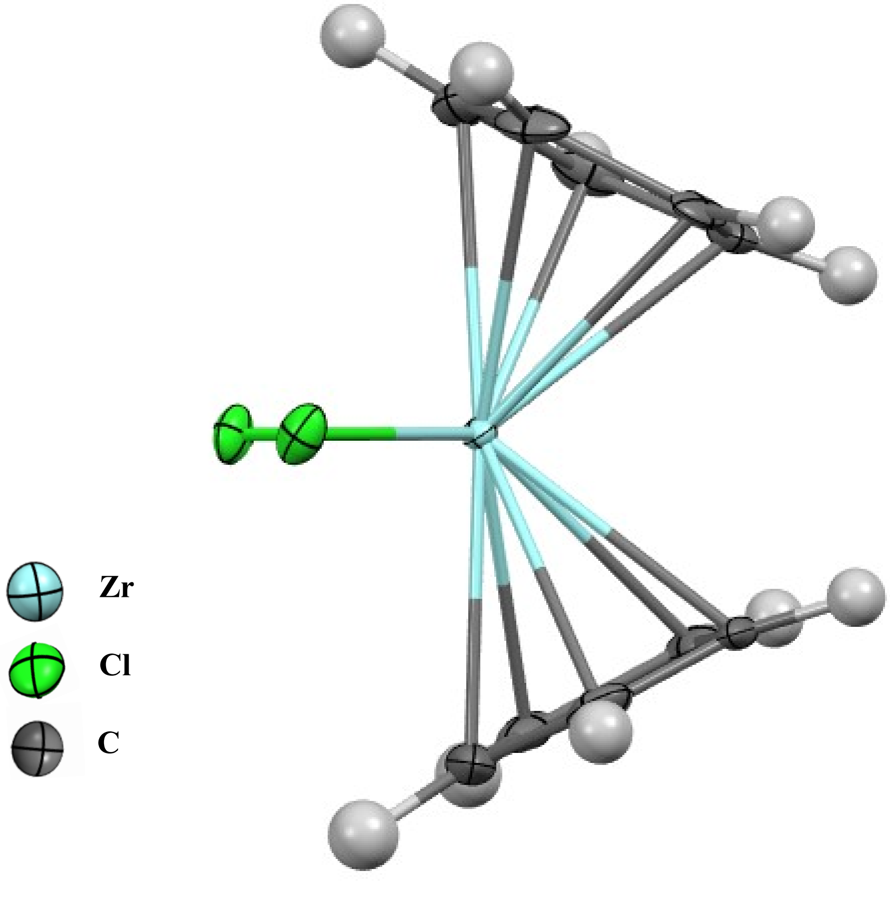

Thermal ellipsoids are displayed at the 50% probability level. S=solvent molecule.

The CCDC number for the crystal reported in this study is 2371314.

$(PPh_3)_3Rh$(2-methylbutyronitrile)BF_4

To a solution of (*S*)-2-methylbutyronitrile (8.4 mg, 0.1 mmol) and sensor **8** (0.1 mmol) in 1.0 mL CH_2Cl_2 was added $AgBF_4$ (20.0 mg, 0.1 mmol) as a solid. The mixture was stirred for 1 hour and subsequently filtered through a 2-micron syringe filter, and 1.0 mL of pentane was carefully layered on top. Red crystals were obtained after 18 hours. Single crystal analysis was performed at 100 K using a Siemens platform diffractometer with a graphite monochromated Mo-Kα radiation (λ = 0.71073 Å). Data were integrated and corrected using the APEX 3 program. The structures were solved by direct methods and refined with full-matrix least-square analysis using SHELXL-2019/1 software. Non-hydrogen atoms were refined with anisotropic displacement parameter. Crystal data: $C_{60}H_{56}BCl_2F_4NP_3Rh$, M= 1144.58, 0.552 x 0.153 x 0.042 mm^3, monoclinic, space group C_2/c, a= 45.401(8), b= 10.5541 (19), c= 25.293(5) Å, Z = 8.

Scheme S10. X-ray crystallographic analysis of the reaction product of (*S*)-2-methylbutyronitrile and **8** in the presence of 1.0 eq $AgBF_4$.

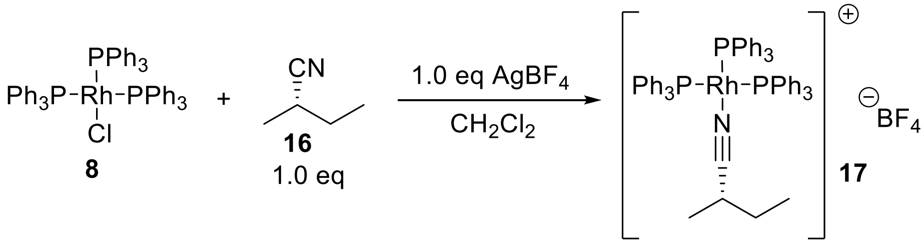

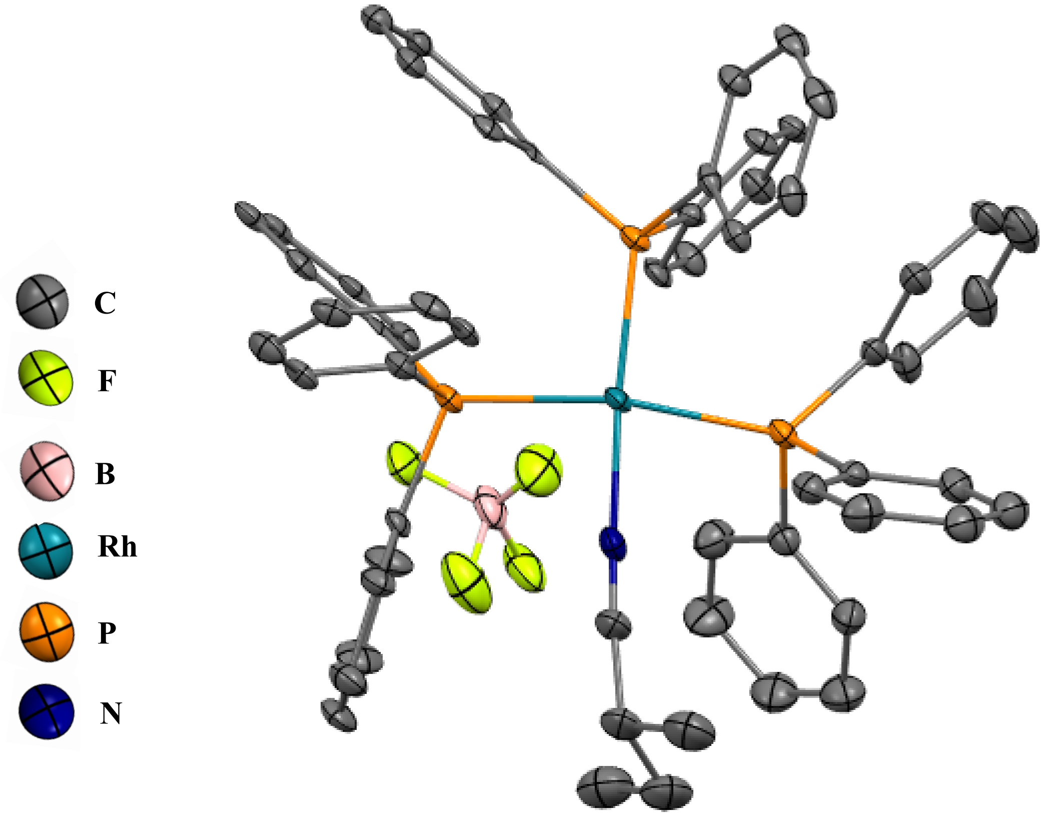

Thermal ellipsoids are displayed at the 50% probability level. Hydrogen atoms and the solvent molecule were removed for clarity.

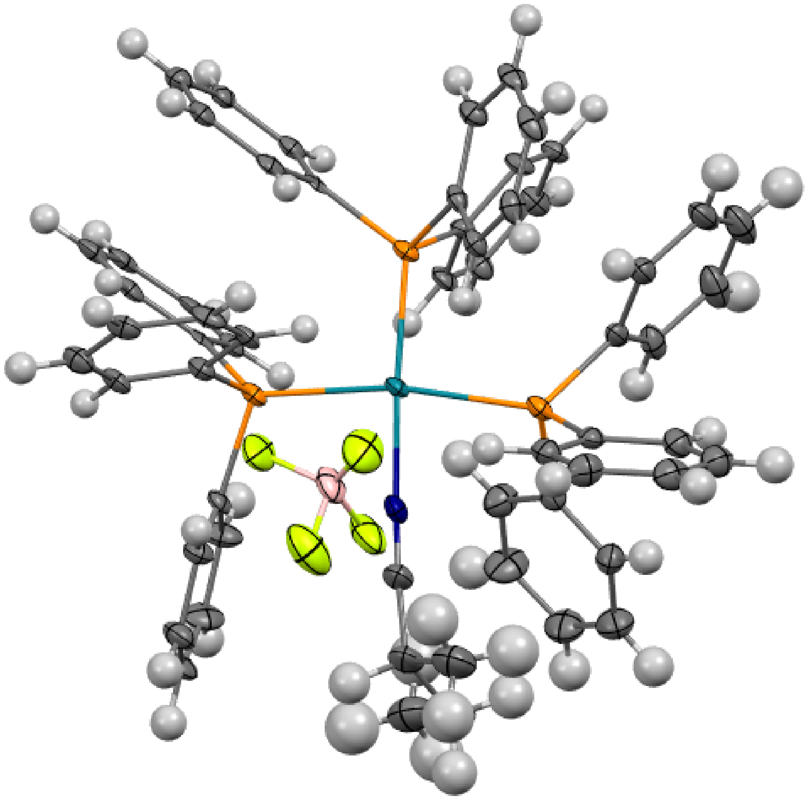

Thermal ellipsoids are displayed at the 50% probability level. The solvent molecule was removed for clarity.

The CCDC number for the crystal reported in this study is 2371315.

3.3. Mass spectrometry

To a solution of (*S*)-2-(5-bromothiophen-2-yl)heptanenitrile (10.0 mg, 0.04 mmol) in 1.0 mL CH_2Cl_2 was added zirconocene chloride hydride (9.5 mg, 0.04 mmol) as a solid. The mixture was stirred for 5 minutes, after which sensor **3** was added (11.6 mg, 0.02 mmol) in 1.5 mL CH_2Cl_2. The mixture was stirred for 18 hours. Direct injection into a single quadrupole LC/MSD iQ using acetonitrile as solvent showed successful transmetalation to sensor **3**.

Figure S12. MS analysis of the transmetalation reaction of (*S*)-2-(5-bromothiophen-2-yl)heptanenitrile to **3** after hydrozirconation.

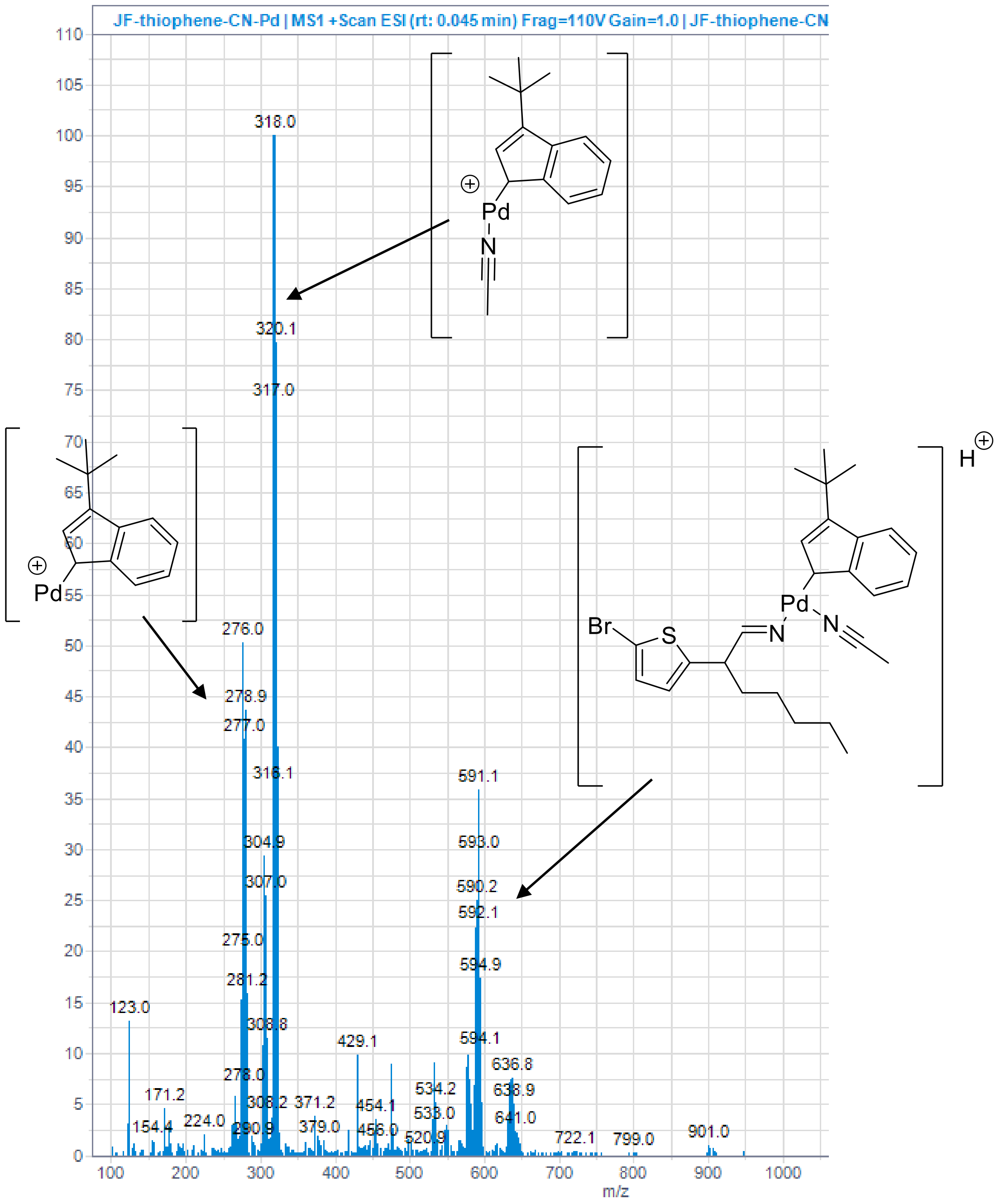

4. Nitrile substrate scope

Scheme S11. Chiral nitrile substrate scope

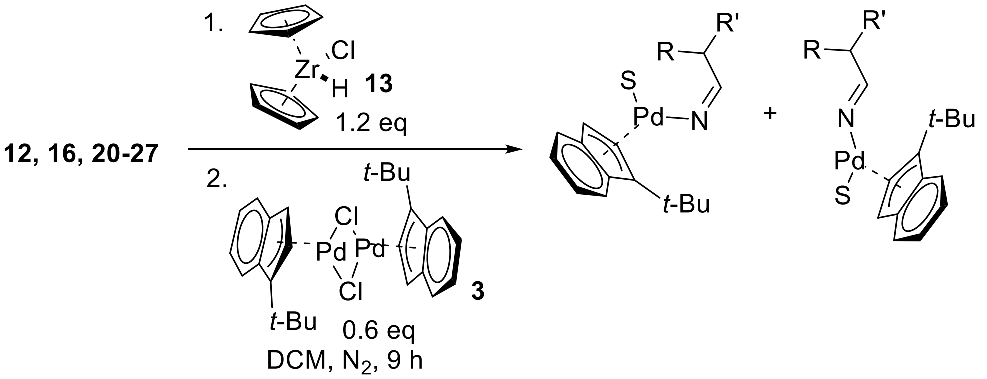

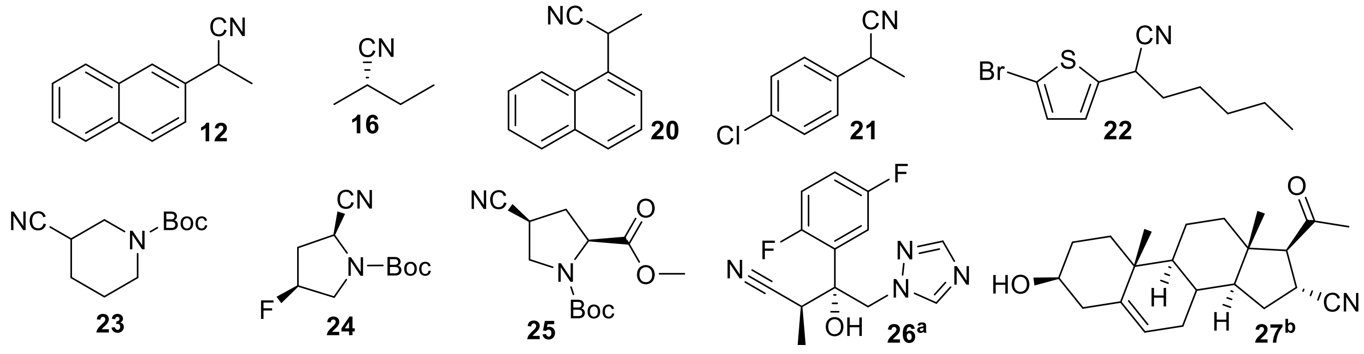

[a] 2.2 eq of **13** and 1.2 eq of **3** were used.
[b] 3.2 eq of **13** and 1.7 eq of **3** were used.
S=solvent

A solution of a chiral nitrile (0.025 mmol) in 1.0 mL of CH_2Cl_2 was added to a vial, followed by the addition of **13** (7.7 mg, 0.03 mmol). The suspension was stirred for 5 minutes, after which the solution turned yellow. The remaining solid was allowed to settle to the bottom of the vial. A 0.2 mL aliquot was taken and added to a new vial, into which a solution of **3** (1.88 mg, 0.003 mmol) in 1.0 mL of DCM was added. The mixture was allowed to stir for 9 hours and then subjected to CD analysis after dilution with DCM to the final concentration indicated under each figure.

Figure S13. CD spectra obtained by applying sensor **3** to (*R*)-**12** (red) and (*S*)-**12** (blue).

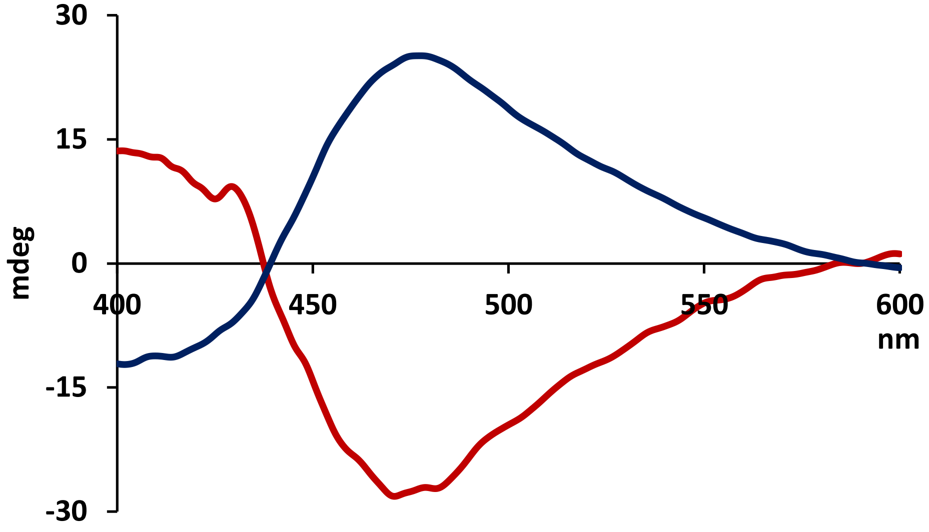

CD measurements were taken at 0.75 mM in CH_2Cl_2.

Figure S14. CD spectra obtained by applying sensor **3** to (*S*)-**16.**

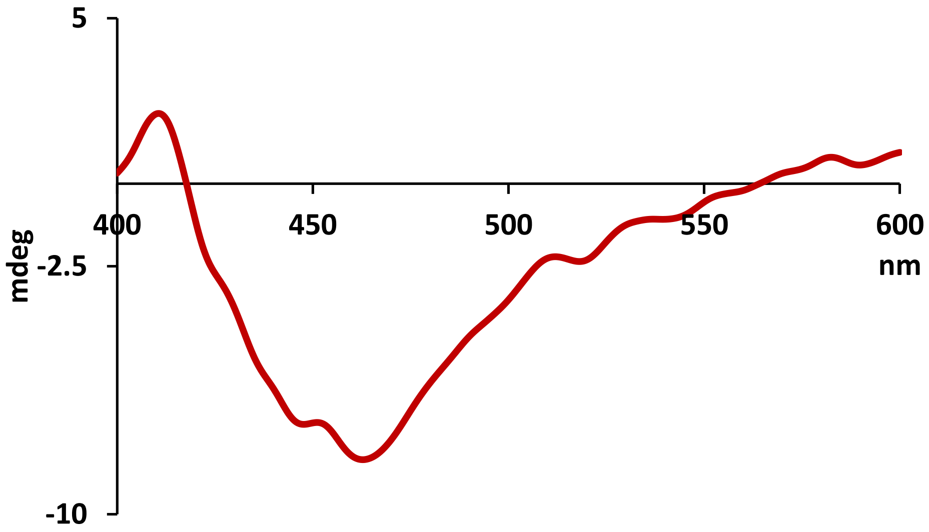

CD measurements were taken at 0.75 mM in CH_2Cl_2.

Figure S15. CD spectra obtained by applying sensor to **3** (*R*)-**20** (red) and (*S*)-**20** (blue).

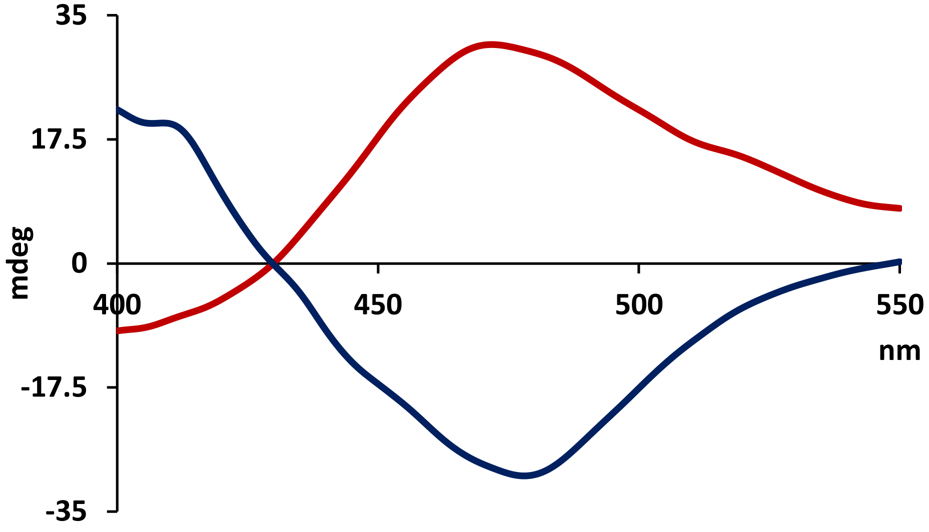

CD measurements were taken at 0.75 mM in CH_2Cl_2.

Figure S16. CD spectra obtained by applying sensor **3** to (*R*)-**21** (red) and (*S*)-**21** (blue).

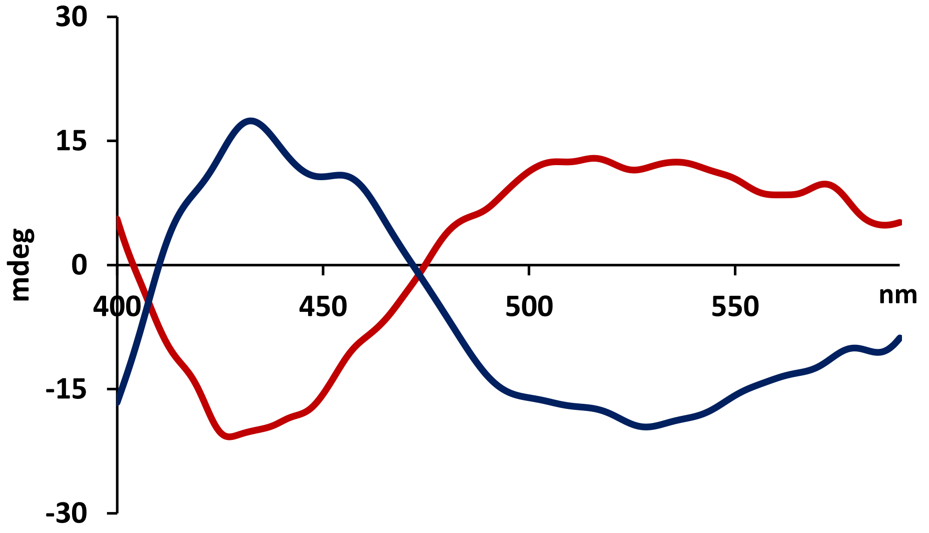

CD measurements were taken at 0.75 mM in CH_2Cl_2.

Figure S17. CD spectra obtained by applying sensor **3** to (*R*)-**22** (red) and (*S*)-**22** (blue)**.**

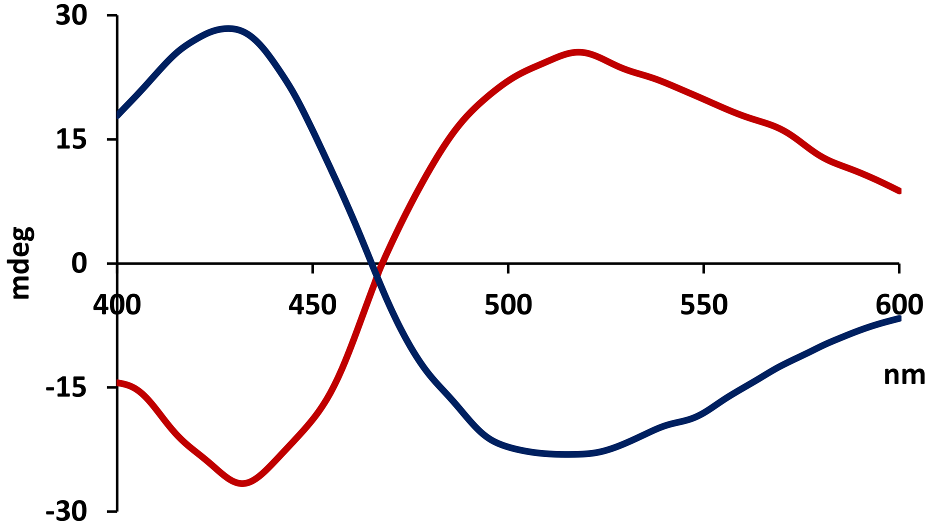

CD measurements were taken at 0.75 mM in CH_2Cl_2.

Figure S18. CD spectra obtained by applying sensor **3** to (*R*)-**23** (red) and (*S*)-**23** (blue).

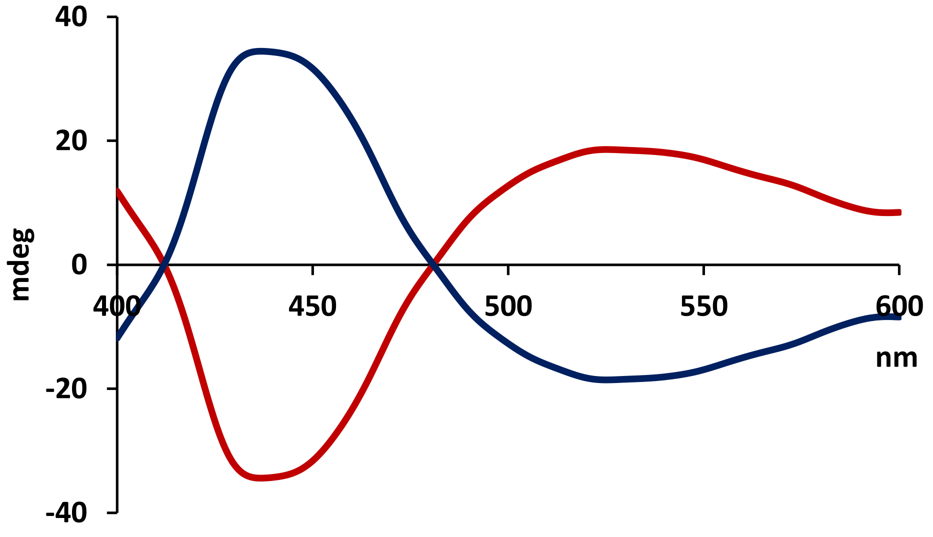

CD measurements were taken at 0.75 mM in CH_2Cl_2.

Figure S19. CD spectra obtained by applying sensor **3** to (*S*,*S*)-**24.**

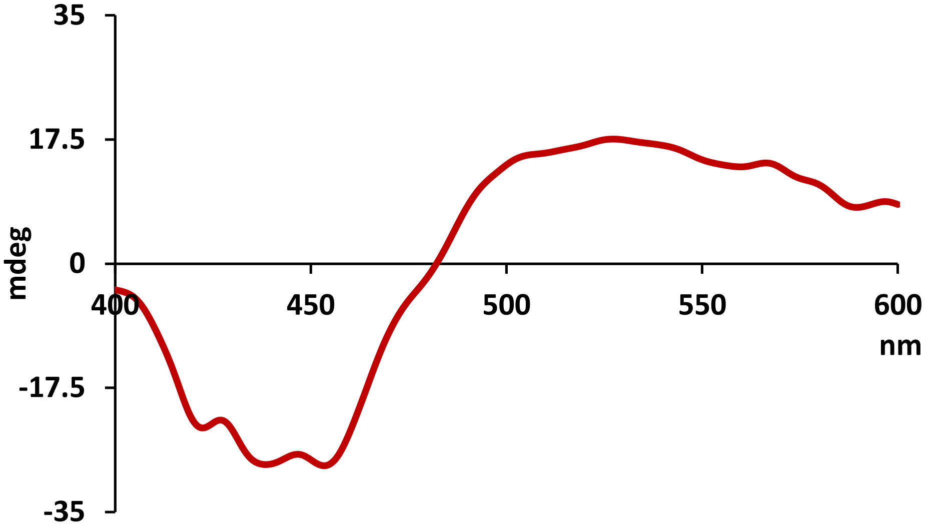

CD measurements were taken at 0.75 mM in CH_2Cl_2.

Figure S20. CD spectra obtained by applying sensor **3** to (*S*,*S*)-**25.**

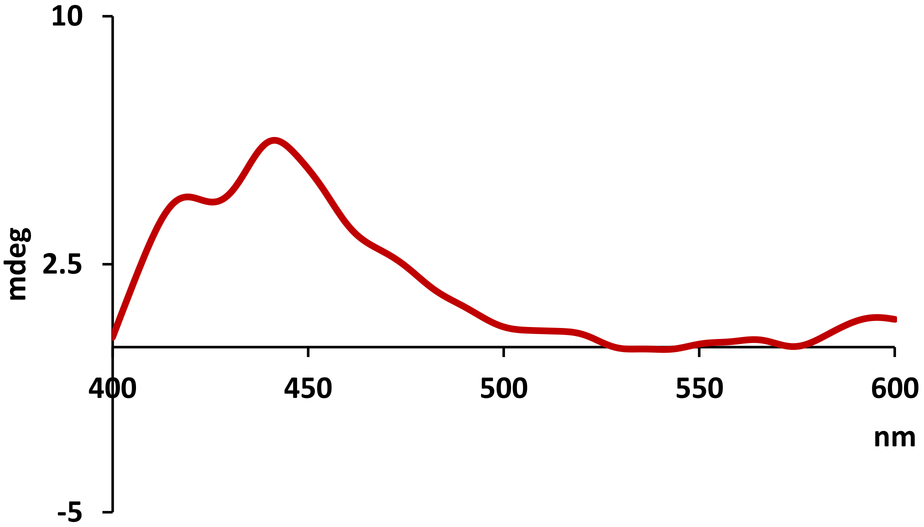

CD measurements were taken at 0.75 mM in CH_2Cl_2.

Figure S21. CD spectra obtained by applying sensor **3** to (*S*,*R*)-**26.**

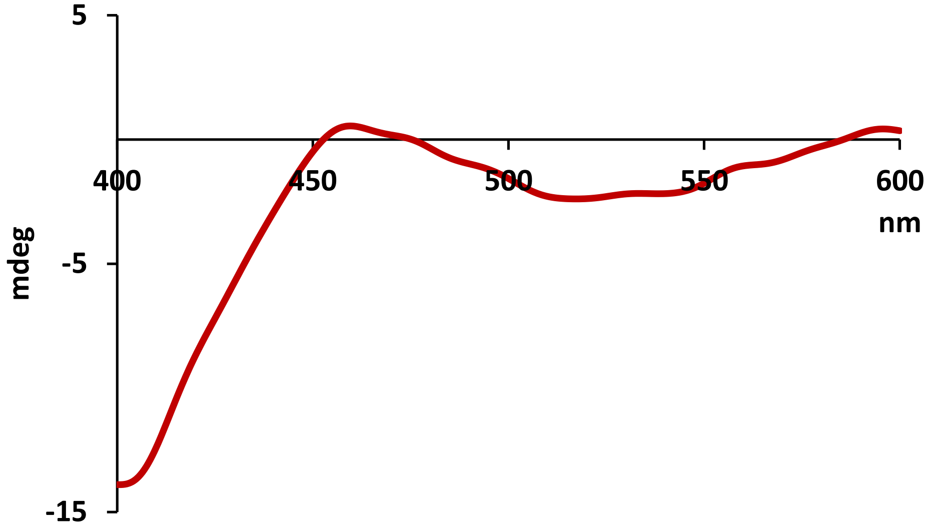

CD measurements were taken at 0.75 mM in CH_2Cl_2.

Figure S22. CD spectra obtained by applying sensor **3** to **27.**

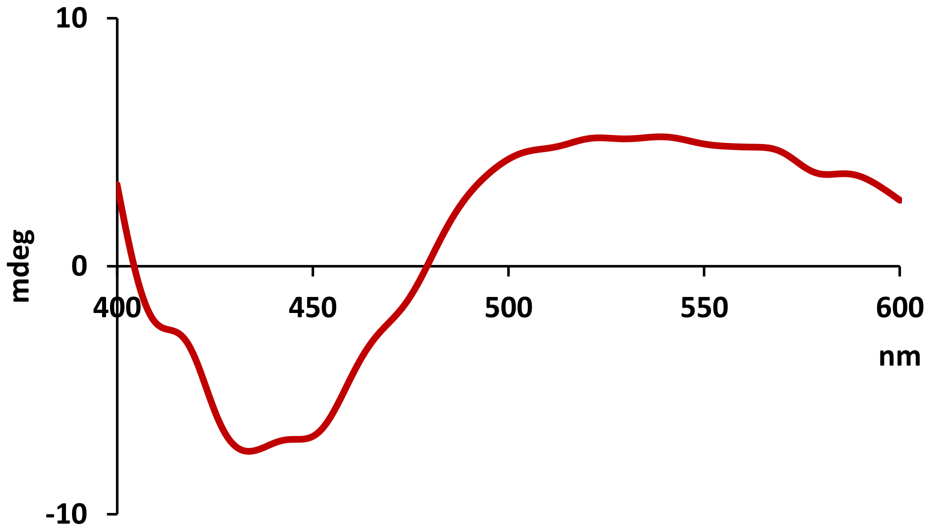

CD measurements were taken at 0.75 mM in CH_2Cl_2.

Because the heterocyclic moieties in analytes **25** and **26** could, in principle, also coordinate to the Pd center we investigated if such an interaction could at least in part contribute to the observed CD inductions. However, both UV and CD experiments didn't reveal any optical changes which supports that the hydrozirconation of the nitrile group prior to the transmetalation step is essential to generate a CD-active palladium complex.

Into an 8 mL vial was added either **25** (12.8 mg, 0.05 mmol) or **26** (13.9 mg, 0.05 mmol), followed by sensor **3** (15.7, 0.025 mmol) and the mixtures were dissolved in 2.0 mL CH_2Cl_2. After stirring for 3 hours, the solutions were subjected to UV and CD analysis. No significant change in the UV spectra was recorded and no CD signal inductions were observed.

Figure S23. UV spectra of **3** and the solutions with analytes **25** or **26**.

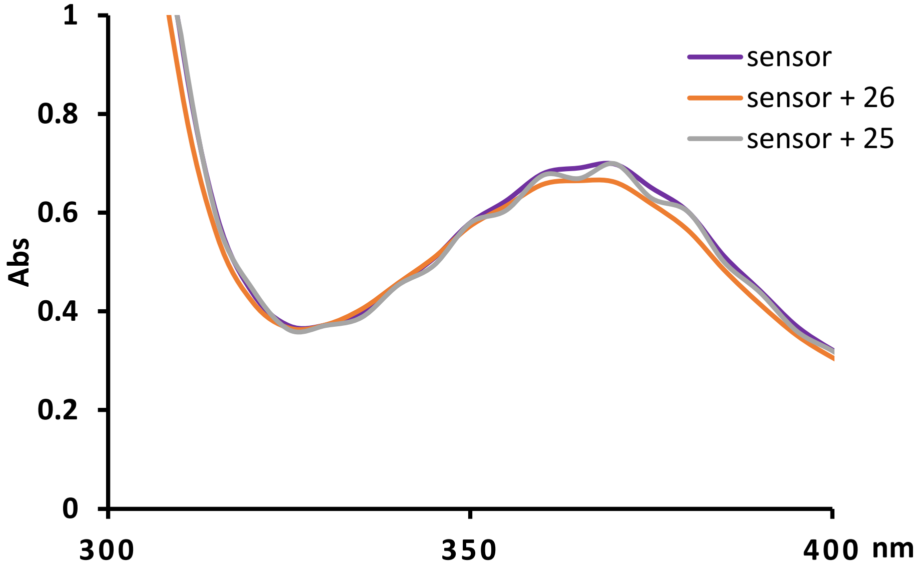

Figure S24. CD measurements of the solution of **3** and **25**.

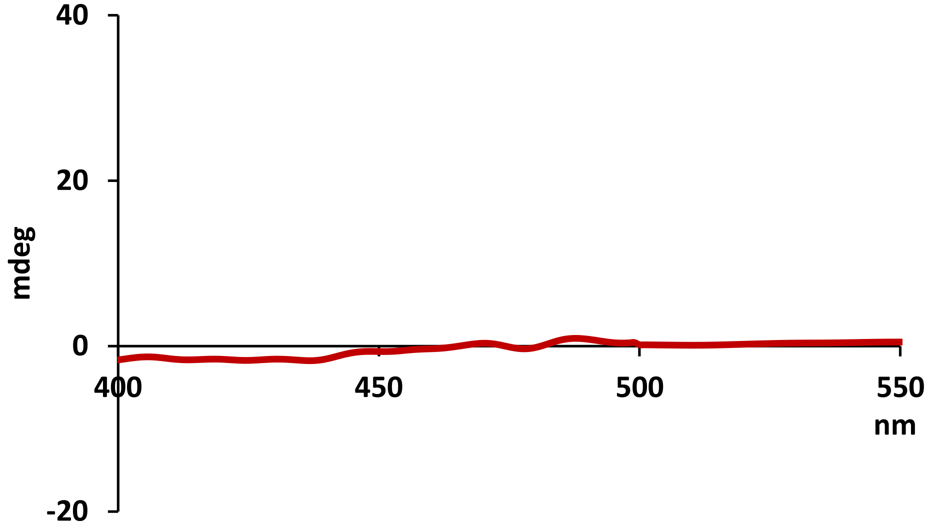

CD measurements were taken at 0.75 mM in CH_2Cl_2.

Figure S25. CD measurements of the solution of **3** and **26**.

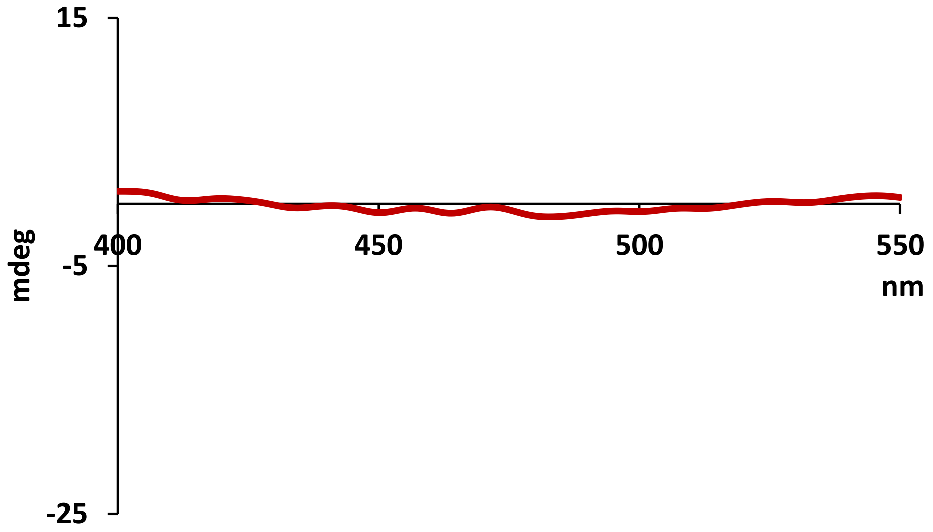

CD measurements were taken at 0.75 mM in CH_2Cl_2.

5. Quantitative nitrile sensing: absolute configuration, enantiomeric excess, and total concentration

The change in UV absorbance upon addition of sensor **3** to the hydrozirconation product of **12** and zirconocene chloride hydride was measured. Stock solutions of sensor **3** (12.5 mM in CH_2Cl_2) and **12** (25.0 mM in CH_2Cl_2) were prepared. To a series of 8 mL vials was added **12** in varying concentrations (0.0, 6.0, 13.0, 19.0, 25.0 mM), followed by the addition of 7.7 mg zirchonocene chloride hydride. The total reaction volume of each vial was 1.0 mL. After 5 minutes, the remaining solid was allowed to settle to the bottom of each vial. A 200.0 µl aliquot was taken from each vial and added to a new vial containing 400.0 µl of **3** (12.5 mM in CH_2Cl_2). The mixture was stirred for 9 hours followed by subjection to UV analysis (50.0 µl aliquot added to 2.0 mL CH_2Cl_2). Plotting the intensity at 370 nm versus each concentration of **12** yielded an linear relation with R^2= 0.998 and y = -0.0092x + 0.9777.

Figure S26. Change in the UV absorbance upon reaction of sensor **3** and the hydrozirconation product of **12**.

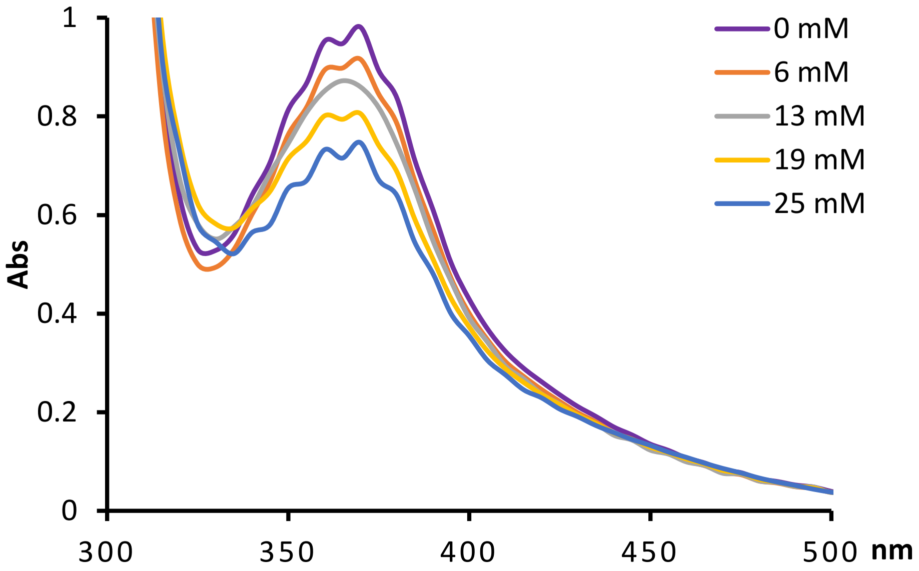

Figure S27. UV absorbance at 370 nm vs. original concentration of **12**.

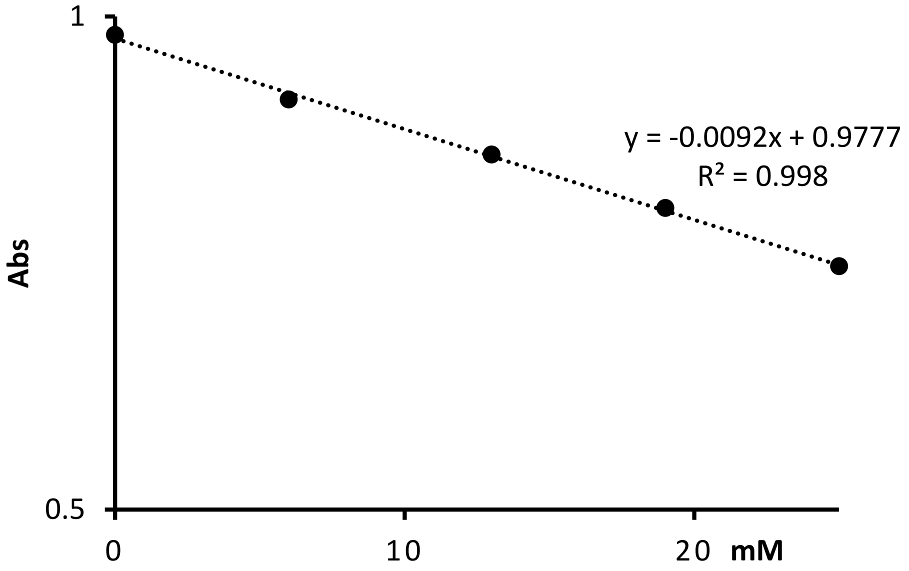

The change in the CD amplitude upon addition of sensor **3** to a solution of zirconocene chloride hydride and **12** was measured. Stock solutions of sensor **3** (12.5 mM in CH_2Cl_2) and **12** (25.0 mM in CH_2Cl_2) were prepared. To a series of 8 mL vials was added **12** (25.0 mM) with varying *ee*'s (+100, +70, +35, -35, -70, -100%), by the addition of 7.7 mg zirchonocene chloride hydride. The total reaction volume of each vial was 1.0 mL. After 5 minutes, the remaining solid was allowed to settle to the bottom of each vial. A 200.0 µl aliquot was taken from each vial and added to a new vial containing 400.0 µl of **3** (12.5 mM in CH_2Cl_2). The mixture was stirred for 9 hours followed by subjection to CD analysis (250.0 µL aliquot diluted with 2.0 mL of CH_2Cl_2). Plotting the CD amplitude at 475 nm against the enantiomeric excess of **12** yielded a straight line with R^2=0.9927 and y= -0.1178x – 0.0517.

Figure S28. Chiroptical response of sensor **3** to scalemic samples of **12**.

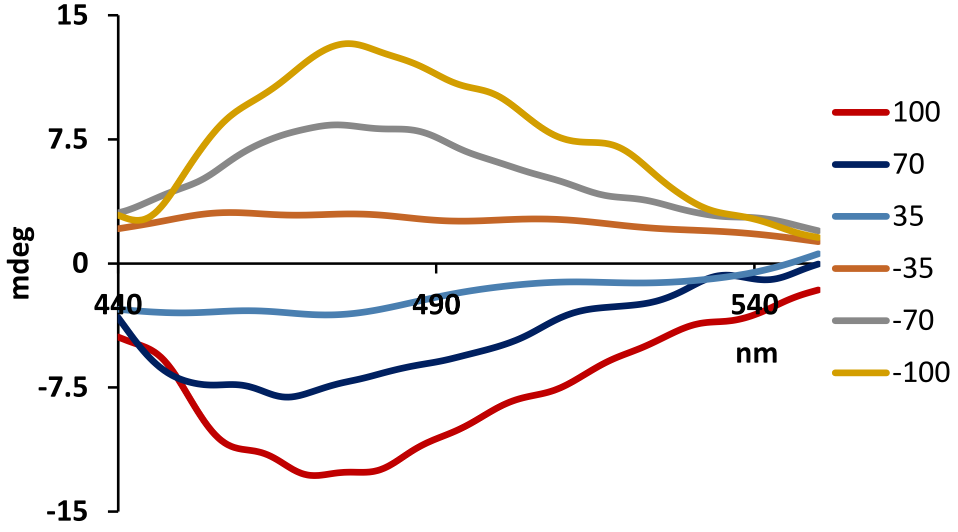

Figure S29. CD amplitude at 475 nm vs sample *ee*.

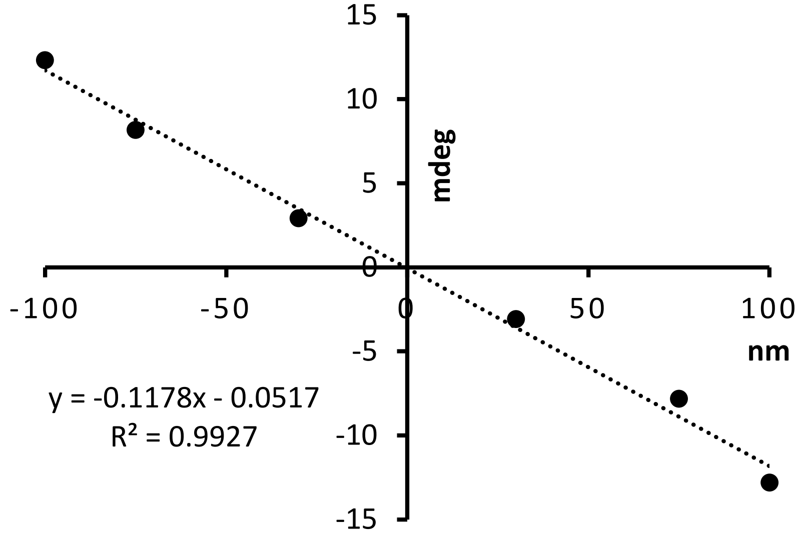

Simultaneous determination of concentration and enantiomeric excess

Ten samples of **12** at varying concentration and enantiomeric composition in CH_2Cl_2 were prepared and subjected to simultaneous analysis of concentration, enantiomeric excess and absolute configuration using sensor **3**. First, a UV spectrum was obtained as described above and the concentration was calculated using the intensities at 370 nm with the equation shown in Figure S24. Then, a CD spectrum was obtained as described above. The CD intensities were normalized to the concentration obtained from UV analysis and the *er* were calculated using the intensities at 475 nm and the equation shown in Figure S26. The absolute configuration was determined by comparing the sign of the Cotton effect to a reference sample.

Table S1. Concentration, enantiomeric ratio, and absolute configuration of samples of 2-(naphthalen-2-yl)propanenitrile determined by simultaneous UV and CD responses of sensor **3.**

Sample Composition			Sensing Results		
Abs. Config.	Concentration (mM)	***Er*** (*R*:*S*)	Abs. Config.	Concentration (mM)	***Er*** (*R*:*S*)
R	22.50	93.5:6.5	*R*	20.80	97.0:3.0
S	12.50	10.0:90.0	*S*	15.60	13.5:86.5
R	23.75	65.0:35.0	*R*	24.50	61.0:39.0
R	10.00	97.5:2.5	*R*	8.00	98.0:2.0
R	17.50	70.0:30.0	*R*	18.80	66.5:33.4
R	15.00	82.5:17.5	*R*	14.60	82.0:17.0
S	20.00	21.0:79.0	*S*	19.40	21.5:78.5
S	8.00	0.0:100.0	*S*	8.40	1.0:99.0
R	18.00	85.0:15.0	*R*	18.90	79.0:21.0
S	21.00	12.5:87.5	*S*	20.10	8.0:92.0

6. Asymmetric nitrile synthesis

General protocol for chiral nitrile synthesis

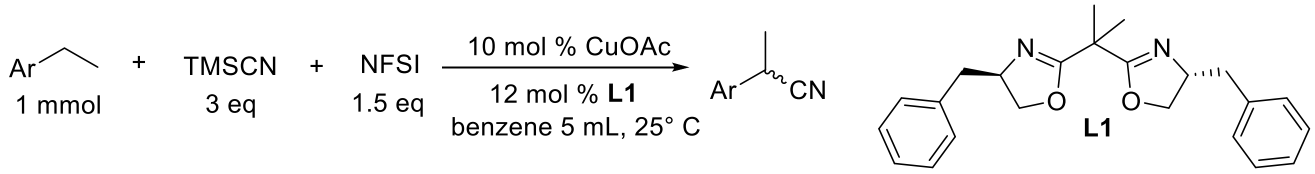

All benzylic nitriles were synthesized using a modified literature protocol.[1] In a 20 mL vial CuOAc (0.01 mmol, 12.4 mg) and **L1** (0.012 mmol, 43.44 mg) were dissolved in benzene (5.0 mL). The mixture was stirred for 30 minutes, after which NFSI (1.5 mmol, 473.01 mg) and substrate (0.1 mmol) were added sequentially. Then, TSMCN (3.0 mmol, 297.6 mg) was added. The reaction was stirred for 18 hours at room temperature. The mixture was washed with water and extracted with ethyl acetate. The combined organic layers were dried over Na_2SO_4 and concentrated *in vacuo*. The desired compound was purified and isolated by flash column chromatography using hexanes – ethyl acetate as the mobile phase.

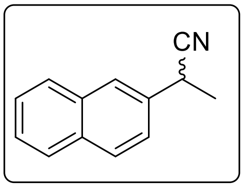

2-(Naphthalen-2-yl)propanenitrile (12). Compound **12** was obtained as a white solid in 70% yield and 95% *ee* (126.7 mg, 0.7 mmol) from 2-ethylnaphthalene (156.2 mg, 1.0 mmol) following the general procedure described above using hexanes/EtOAc (95:5) as the mobile phase. ^{1}H NMR (400 MHz, $CDCl_3$): δ= 7.89-7.80 (m, 4H), 7.55-7.45 (m, 2H), 7.42 (m, 1H), 4.05 (q, J = 7.3 Hz, 1H), 1.72 (d, J = 7.2 Hz, 3H). ^{13}C NMR (100 MHz, $CDCl_3$): δ= 134.3, 133.3, 132.8, 129.1, 127.8, 126.6, 125.6, 125.5, 124.5, 124.3, 121.6, 31.5, 21.8. The spectroscopic data are in accordance with the literature.[1]

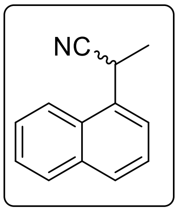

2-(Naphthalen-1-yl)propanenitrile (20). Compound **20** was obtained as a colorless oil in 71% yield and 99% *ee* (126.9 mg, 0.7 mmol) from 1-ethylnaphthalene (156.2 mg, 1.0 mmol) following the general procedure described above using hexanes/EtOAc (95:5) as the mobile phase. ^{1}H NMR (400 MHz, $CDCl_3$): δ= 7.96-7.87 (m, 2H), 7.83 (d, J = 8.3 Hz, 1H), 7.70 (d, J = 7.2 Hz, 1H), 7.63-7.44 (m, 3H), 4.60 (q, J = 7.2 Hz, 1H), 1.76 (d, J = 7.2 Hz, 3H). ^{13}C NMR (100 MHz, $CDCl_3$): δ= 134.0, 132.7, 1329.8, 129.4, 129.0, 126.9, 126.1, 125.5, 124.8, 122.2, 121.9, 28.2, 20.6. The spectroscopic data are in accordance with the literature.[1]

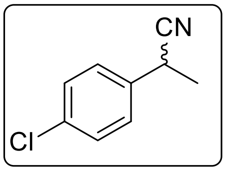

2-(4-Chlorophenyl)propanennitrile (21). Compound **21** was obtained as a colorless oil in 40% yield and 90% *ee* (66.2 mg, 0.4 mmol) from 1-chloro-4-ethylbenzene (140.6 mg, 1.0 mmol) following the general procedure described above using hexanes/EtOAc (95:5) as the mobile phase. ^{1}H NMR (400 MHz, $CDCl_3$): δ= 7.35 (d, J = 8.4 Hz, 2H), 7.28 (d, J = 8.4 Hz, 2H), 3.87 (q, J = 7.3 Hz, 1H), 1.62 (d, J = 7.4 Hz, 3H). ^{13}C NMR (100 MHz, $CDCl_3$): δ= 136.6, 134.1, 129.2, 128.0, 121.7, 30.2, 22.8. The spectroscopic data are in accordance with the literature.[2]

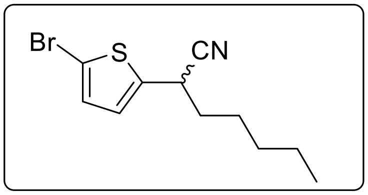

2-(5-Bromothiophen-2-yl)heptanenitrile (22). Compound **22** was obtained as a yellow oil in 60% yield and 99% *ee* (163.3 mg, 0.6 mmol) from 2-bromo-5-hexylthiophene (247.2 mg, 1.0 mmol) following the general procedure described above using hexanes/EtOAc (98:2) as the mobile phase. ^{1}H NMR (400 MHz, $CDCl_3$): δ= 6.91 (d, J = 3.8 Hz, 1H), 6.81 (d, J = 3.8 Hz, 1H), 3.95 (t, J = 7.8 Hz, 1H), 1.97-1.83 (m, 2H), 1.62-1.37 (m, 2H), 1.33-1.26 (m, 4H), 0.87 (m, 3H). ^{13}C NMR (100 MHz, $CDCl_3$): δ= 139.5, 129.8, 126.4, 119.3, 112.0, 35.5, 32.7, 30.9, 26.5, 22.3, 13.9. HRMS (ESI-TOF) *m/z*: (M+), calc'd for $C_{11}H_{14}BrNS$: 271.0030, found: 271.0018.

Figure S30. ^{1}H NMR (400 MHz) spectrum of **12** in $CDCl_3$.

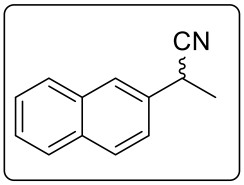

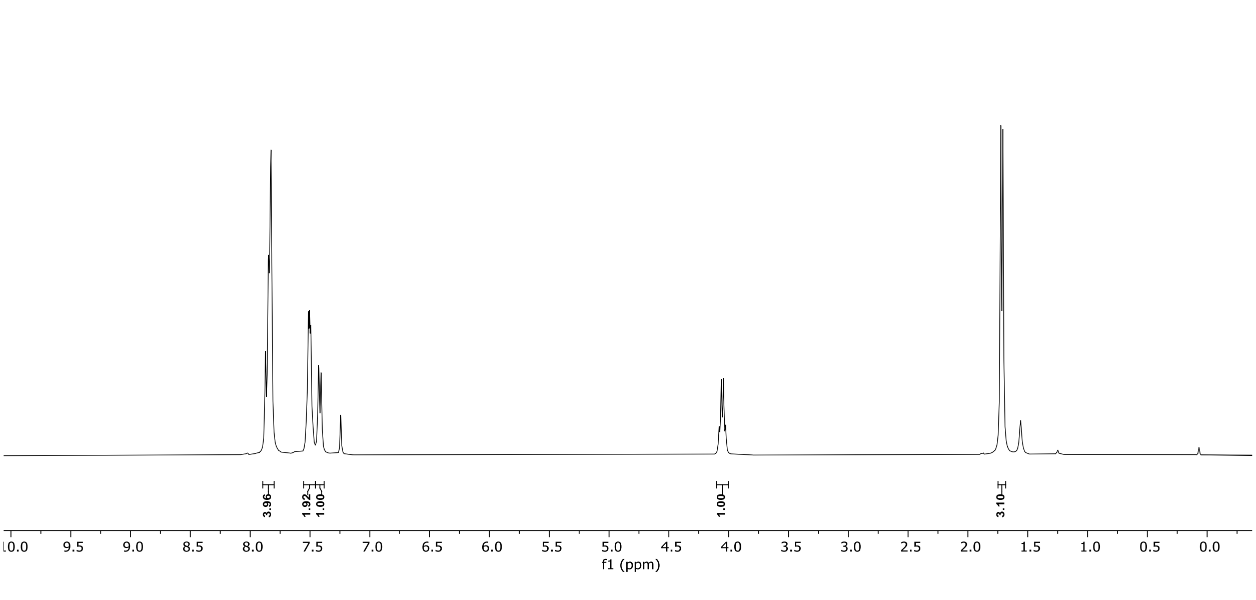

Figure S31. ^{13}C NMR (100 MHz) spectrum of **12** $CDCl_3$.

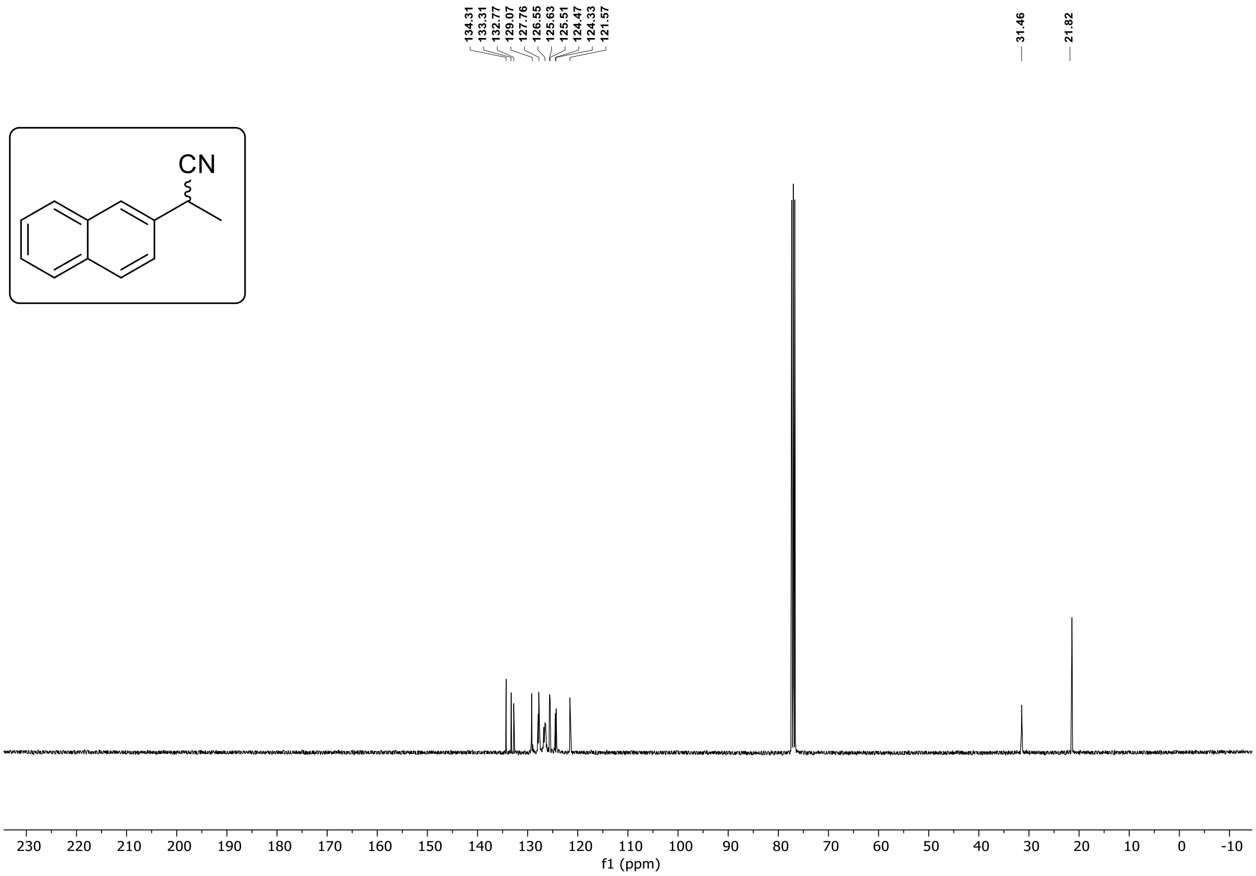

Figure S32. GC-MS of enantioenriched (*R*)-2-(naphthalen-2-yl)propanenitrile (**12**).

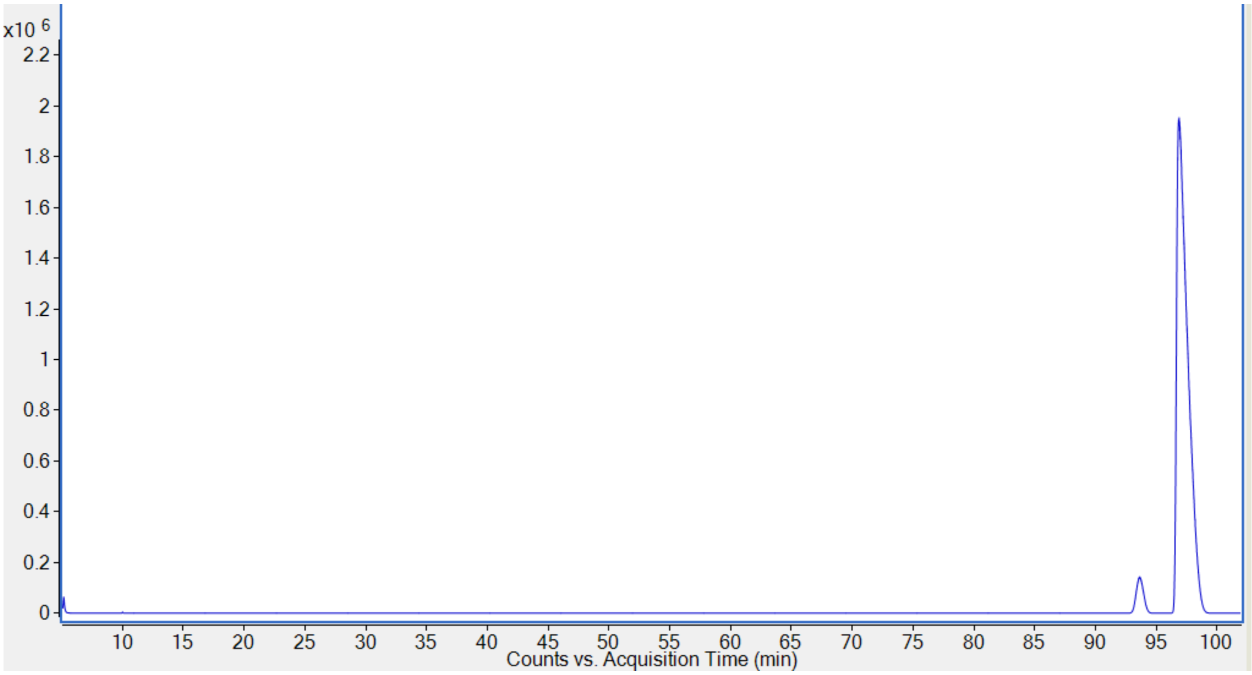

Column: 2,6-dimethyl-3-pentyl-beta-cyclodextrin (50% in polysiloxane), 30 m, 130 °C, pressure: 12.9 psi helium.

Figure S33. GC-MS of enantioenriched (*S*)-2-(naphthalen-2-yl)propanenitrile (**12**).

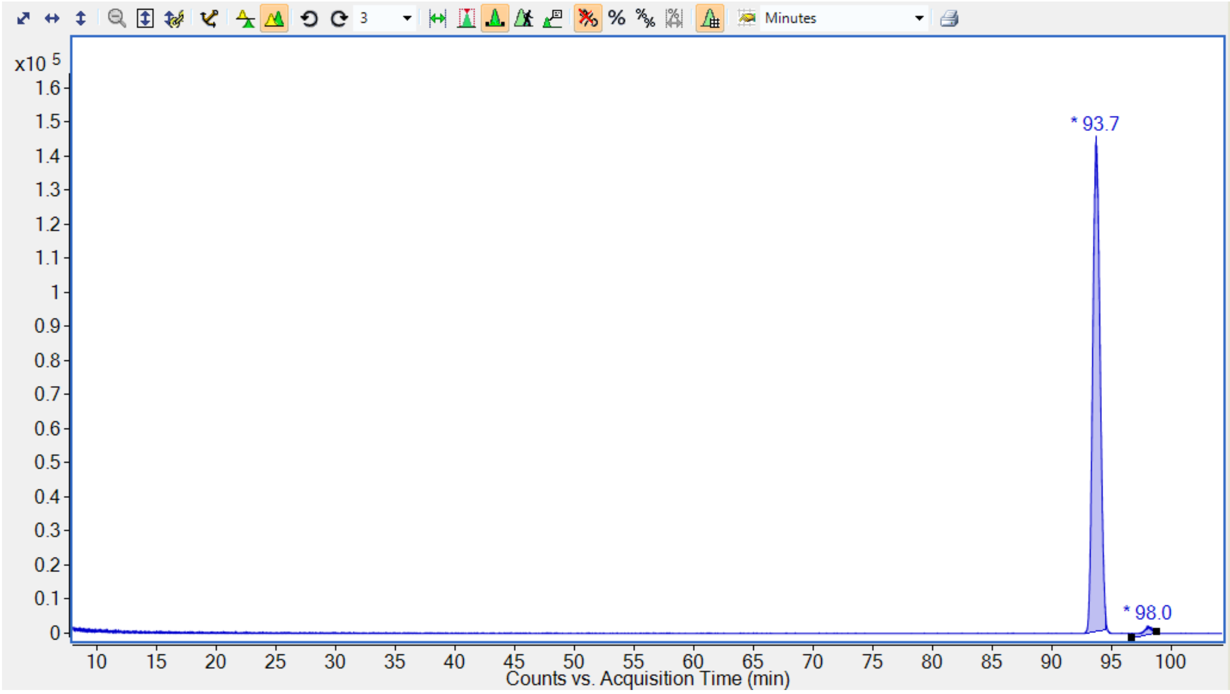

Column: 2,6-dimethyl-3-pentyl-beta-cyclodextrin (50% in polysiloxane), 30 m, 130 °C, pressure: 12.9 psi helium.

Figure S34. ^{1}H NMR (400 MHz) spectrum of **20** in $CDCl_3$.

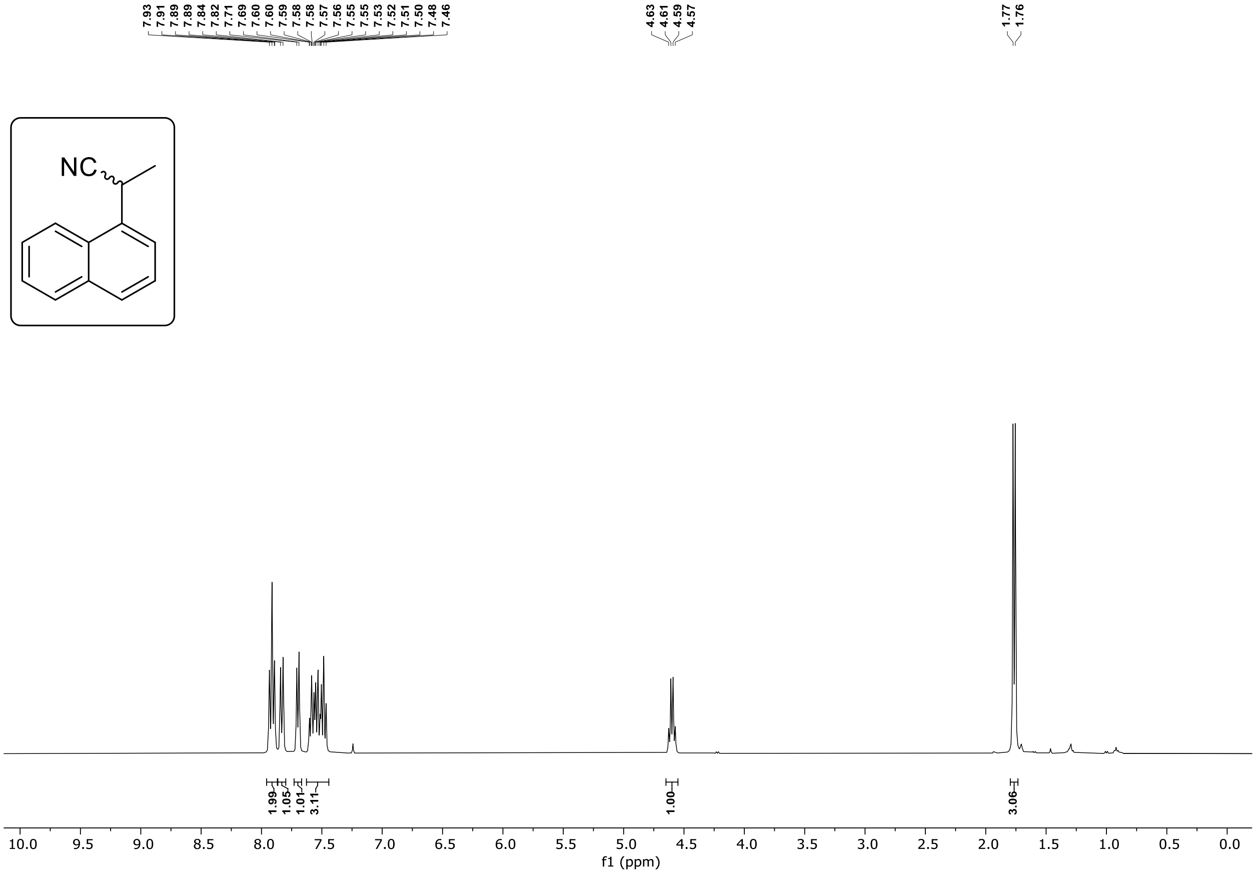

Figure S35. ^{13}C NMR (100 MHz) spectrum of **20** in $CDCl_3$.

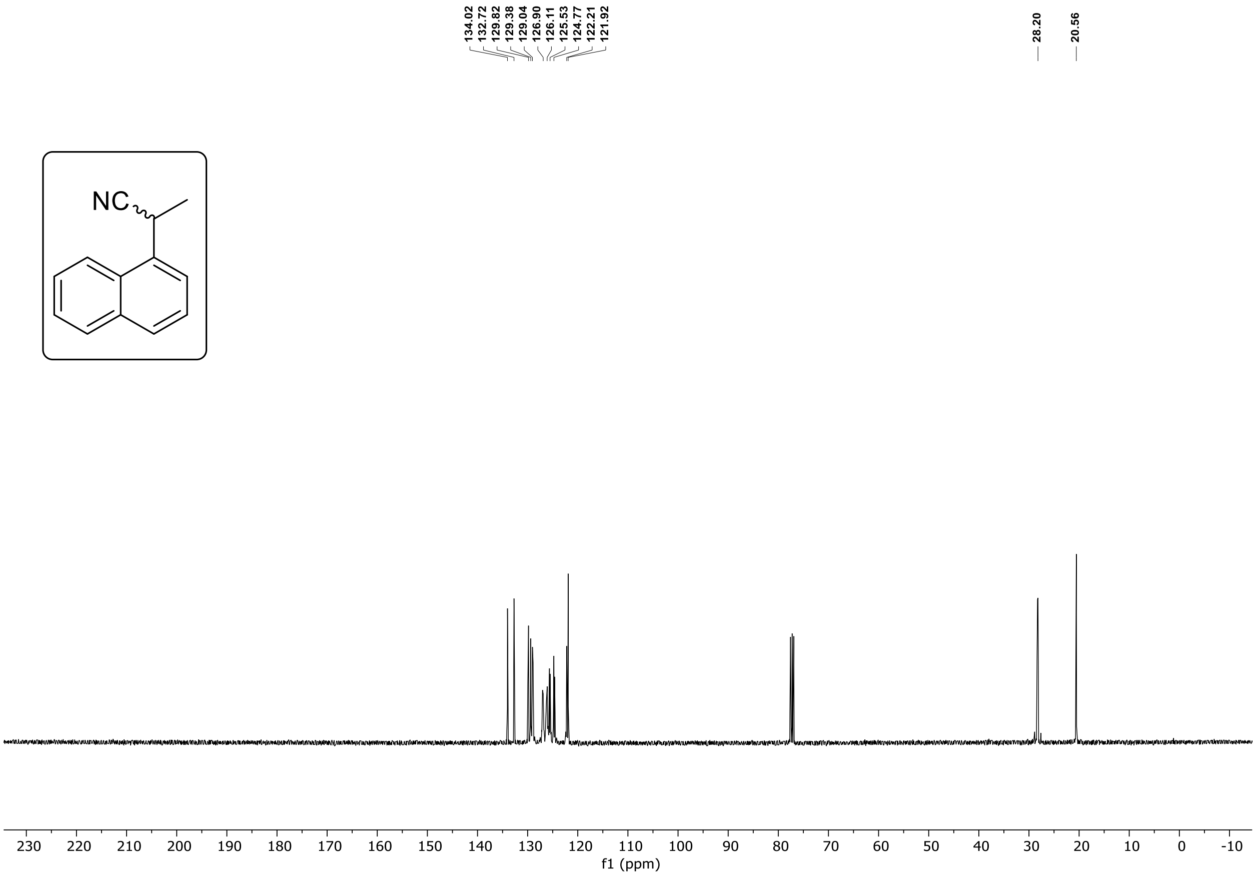

Figure S36. GC-MS of enantioenriched (*R*)-2-(naphthalen-1-yl)propanenitrile (**20**).

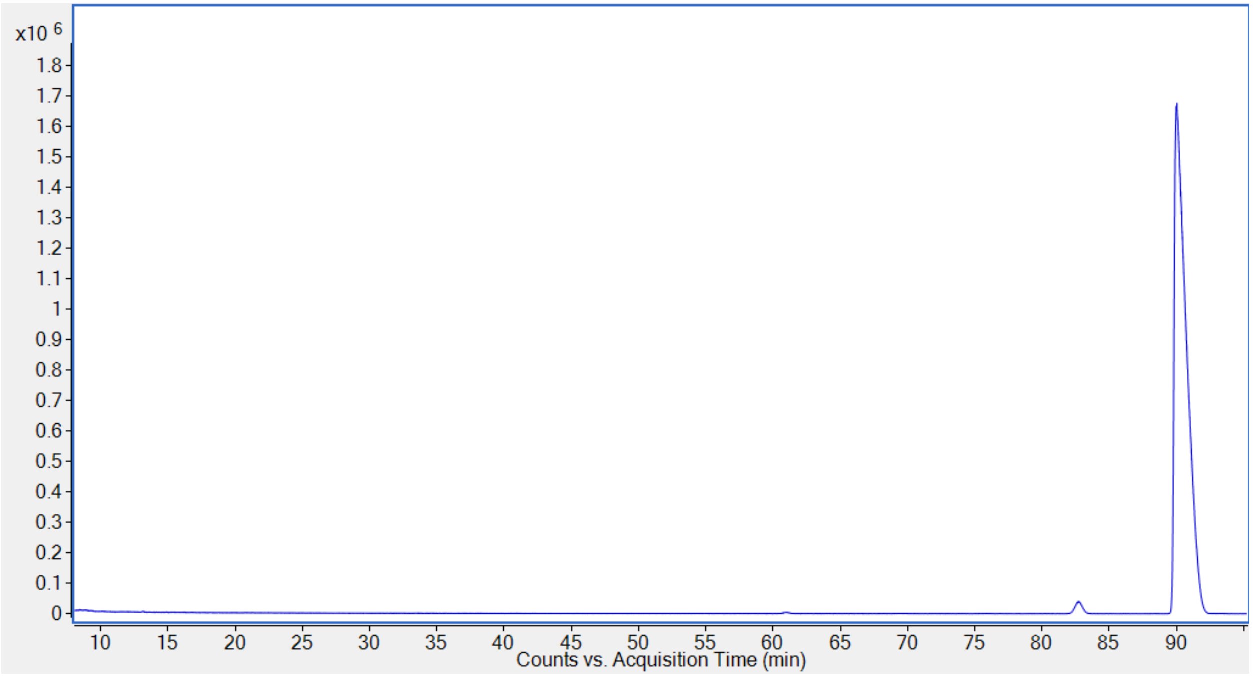

Column: 2,6-dimethyl-3-pentyl-beta-cyclodextrin (50% in polysiloxane), 30 m, 130 °C, pressure: 12.9 psi helium.

Figure S37. GC-MS of enantioenriched (*S*)-2-(naphthalen-1-yl)propanenitrile (**20**).

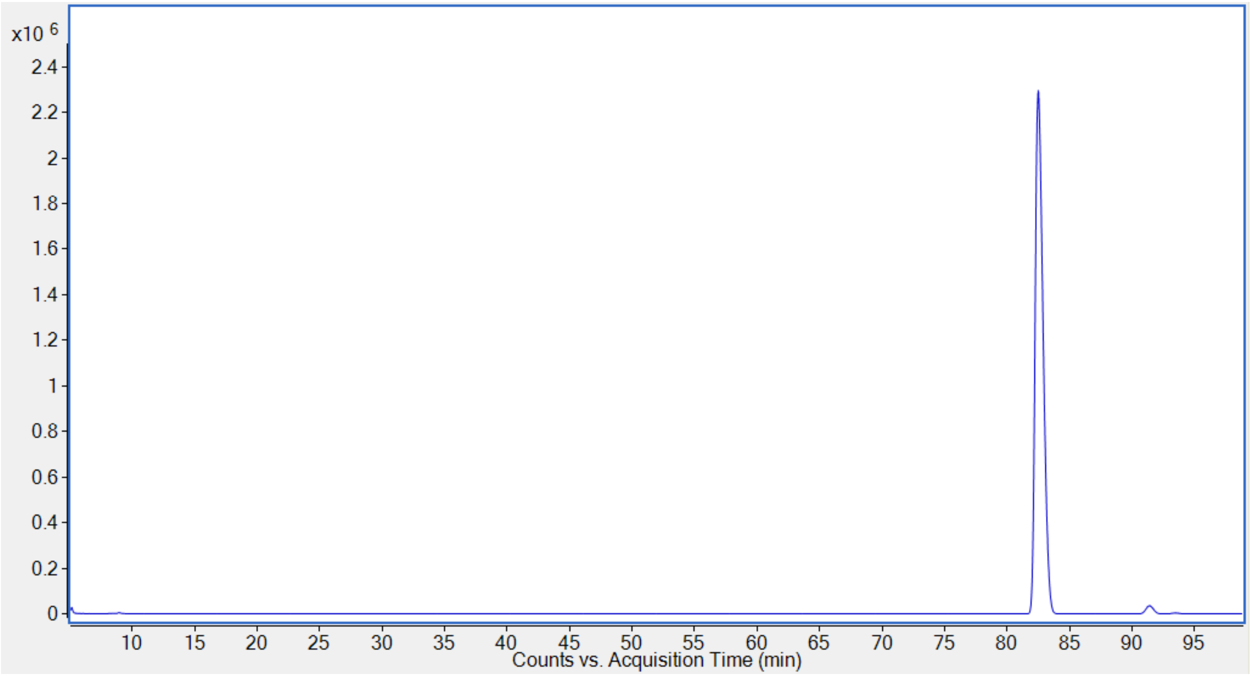

Column: 2,6-dimethyl-3-pentyl-beta-cyclodextrin (50% in polysiloxane), 30 m, 130 °C, pressure: 12.9 psi helium.

Figure S38. ^{1}H NMR (400 MHz) spectrum of **21** in $CDCl_3$.

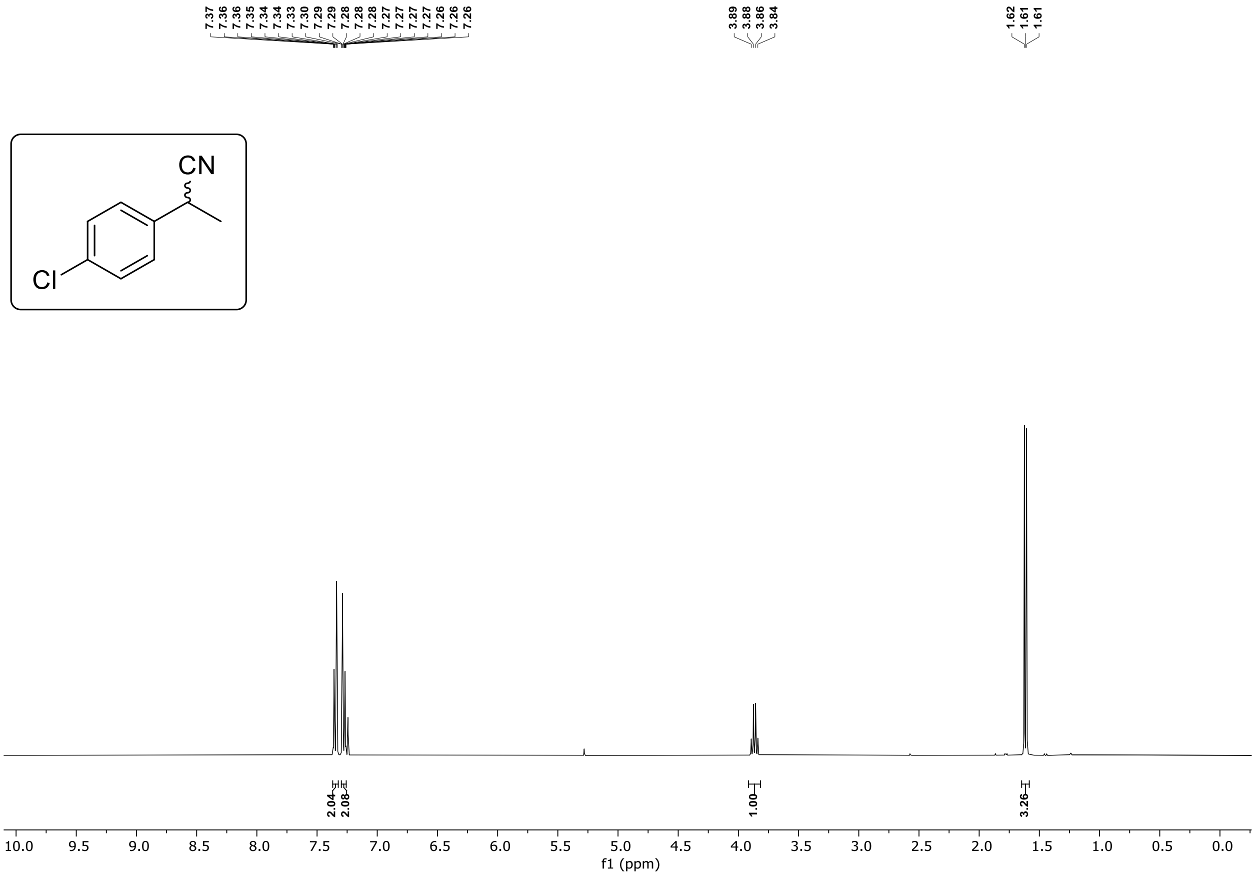

Figure S39. ^{13}C NMR (100 MHz) spectrum of **21** in $CDCl_3$.

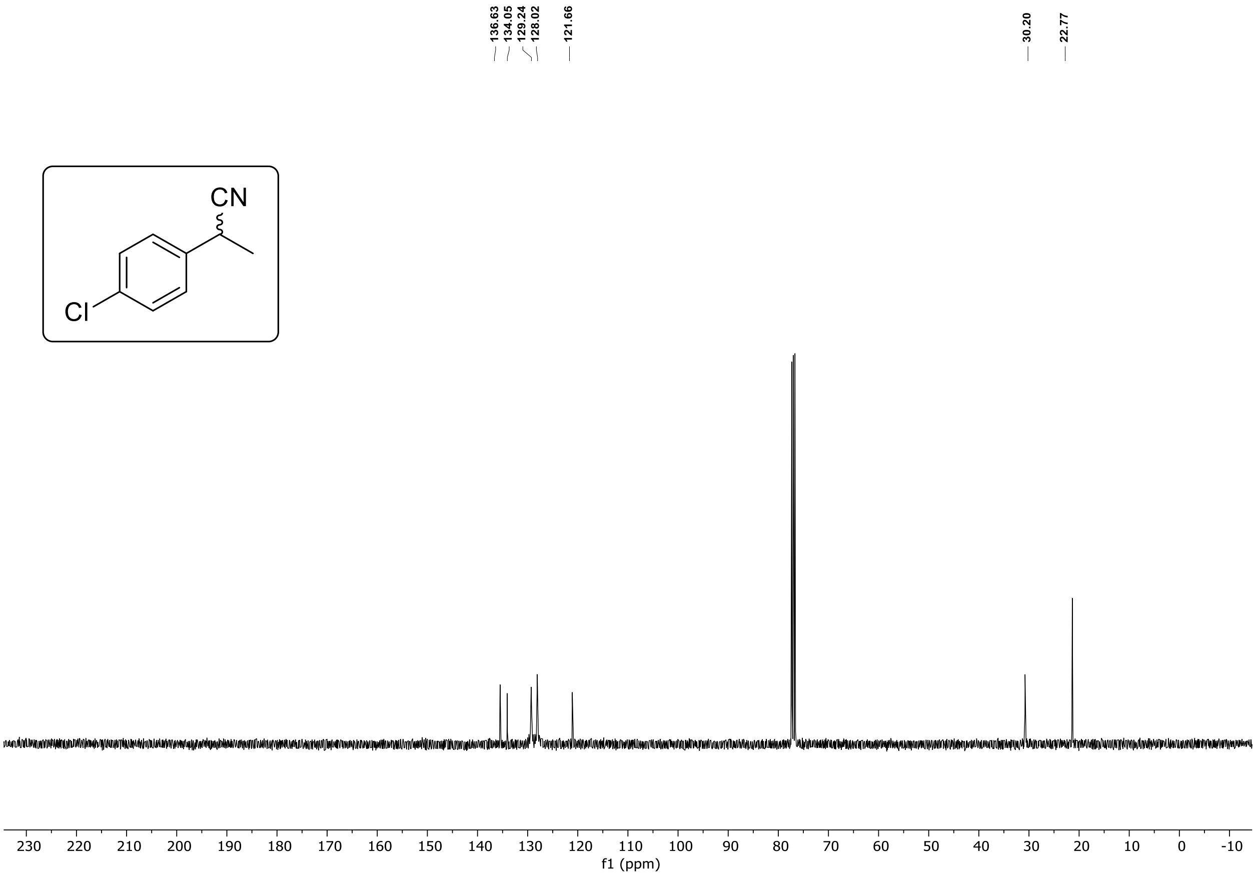

Figure S40. GC-MS of enantioenriched (*R*)-2-(4-chlorophenyl)propanennitrile (**21**).

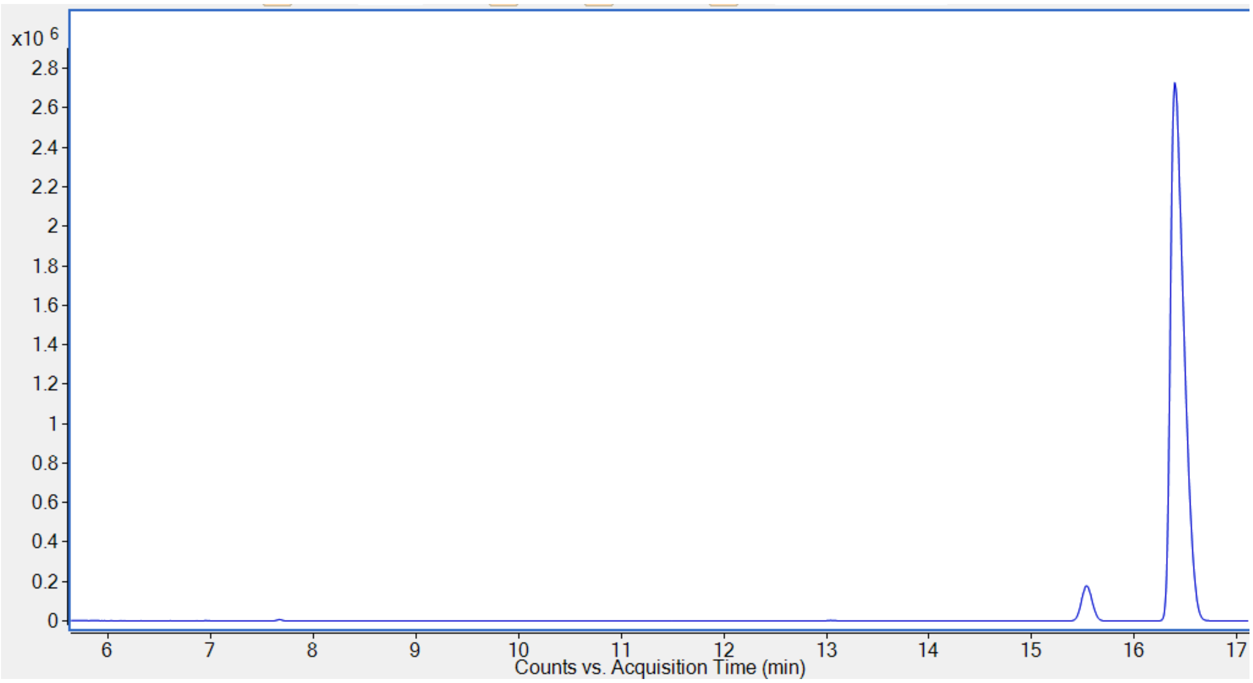

Column: 2,6-dimethyl-3-pentyl-beta-cyclodextrin (50% in polysiloxane), 30 m, 130 °C, pressure: 12.9 psi helium.

Figure S41. GC-MS of enantioenriched (*S*)-2-(4-chlorophenyl)propanennitrile (**21**).

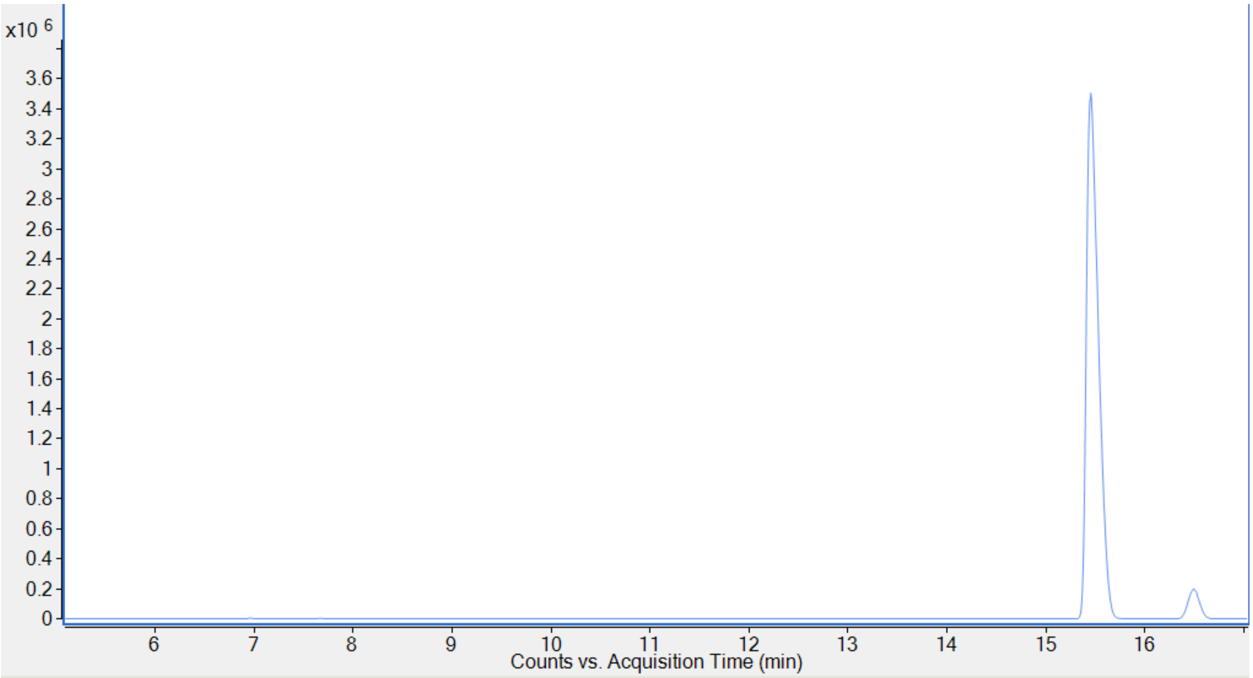

Column: 2,6-dimethyl-3-pentyl-beta-cyclodextrin (50% in polysiloxane), 30 m, 130 °C, pressure: 12.9 psi helium.

Figure S42. ^{1}H NMR (400 MHz) spectrum of **22** in $CDCl_3$.

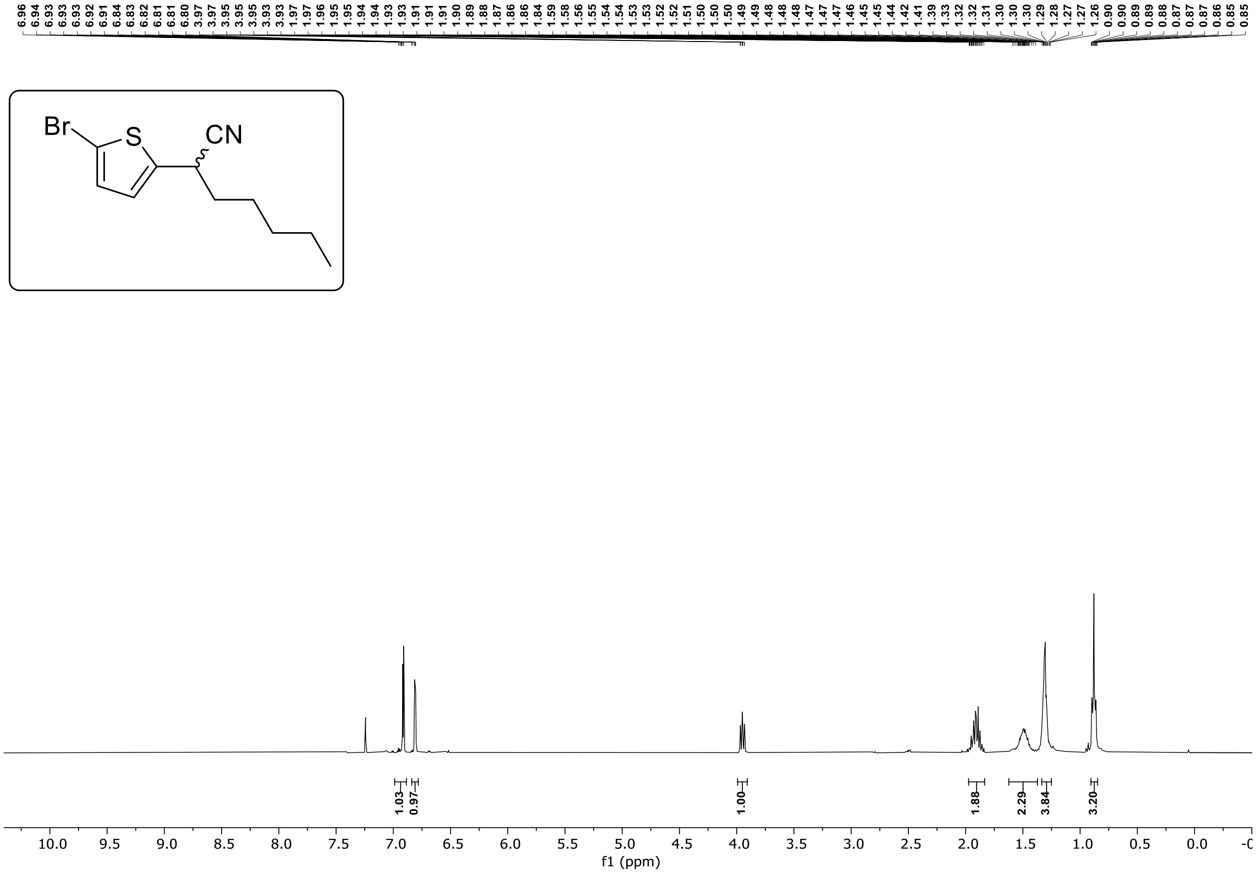

Figure S43. ^{13}C NMR (100 MHz) spectrum of **22** in $CDCl_3$.

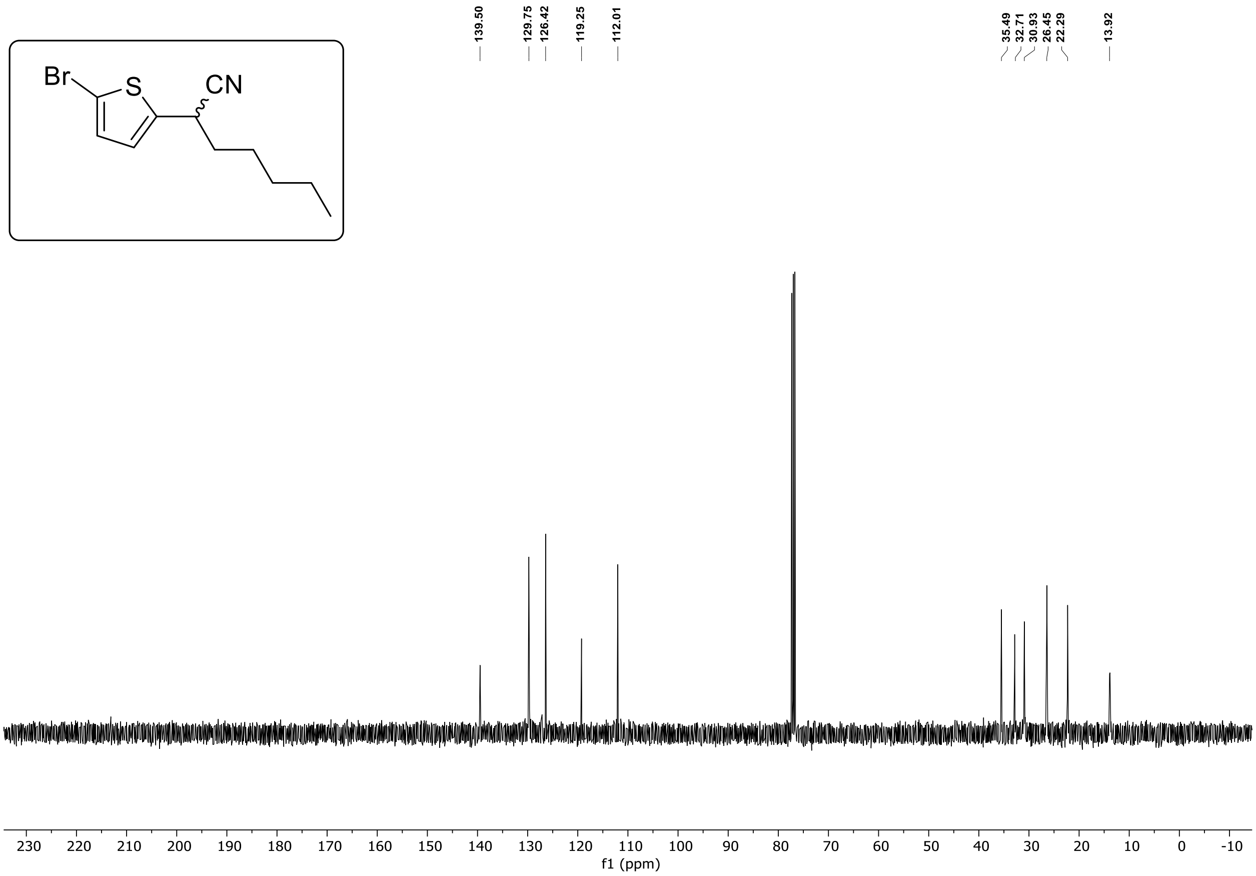

Figure S44. GC-MS of enantioenriched (*R*)- 2-(5-bromothiophen-2-yl)heptanenitrile (**22**).

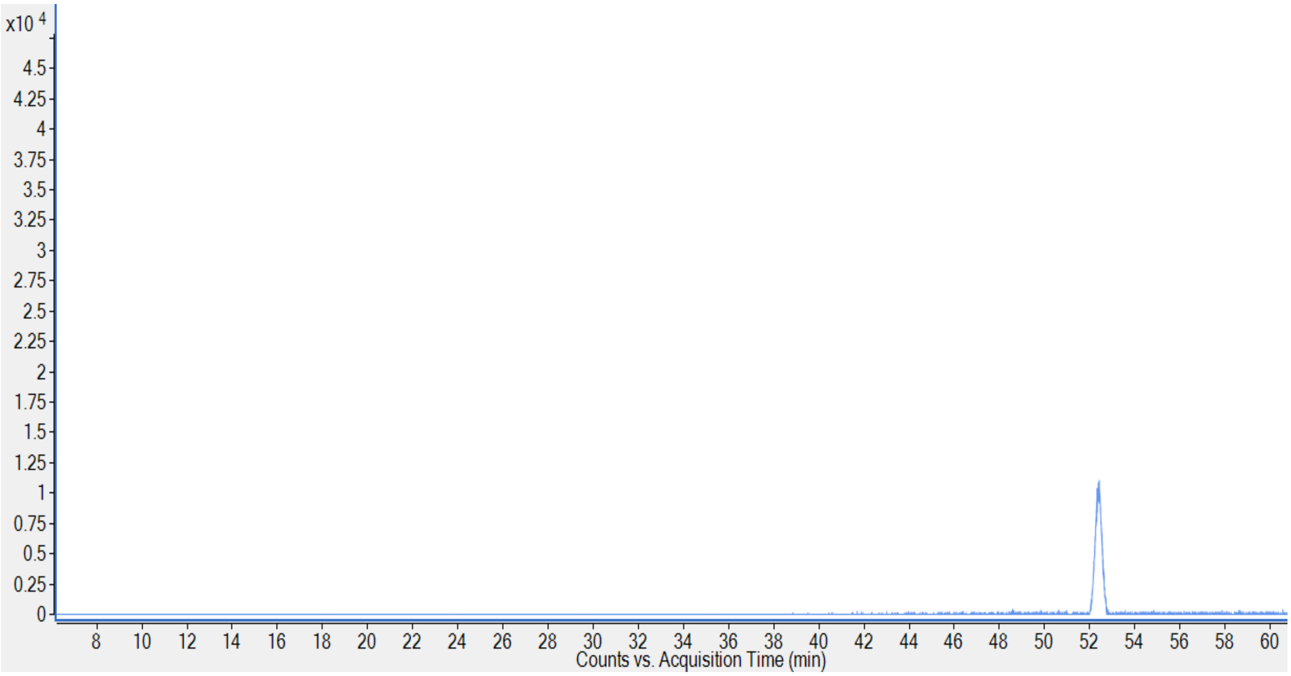

Column: 2,6-dimethyl-3-pentyl-beta-cyclodextrin (50% in polysiloxane), 30 m, 130 °C, pressure: 12.9 psi helium.

Figure S45. GC-MS of enantioenriched (*S*)- 2-(5-bromothiophen-2-yl)heptanenitrile (**22**).

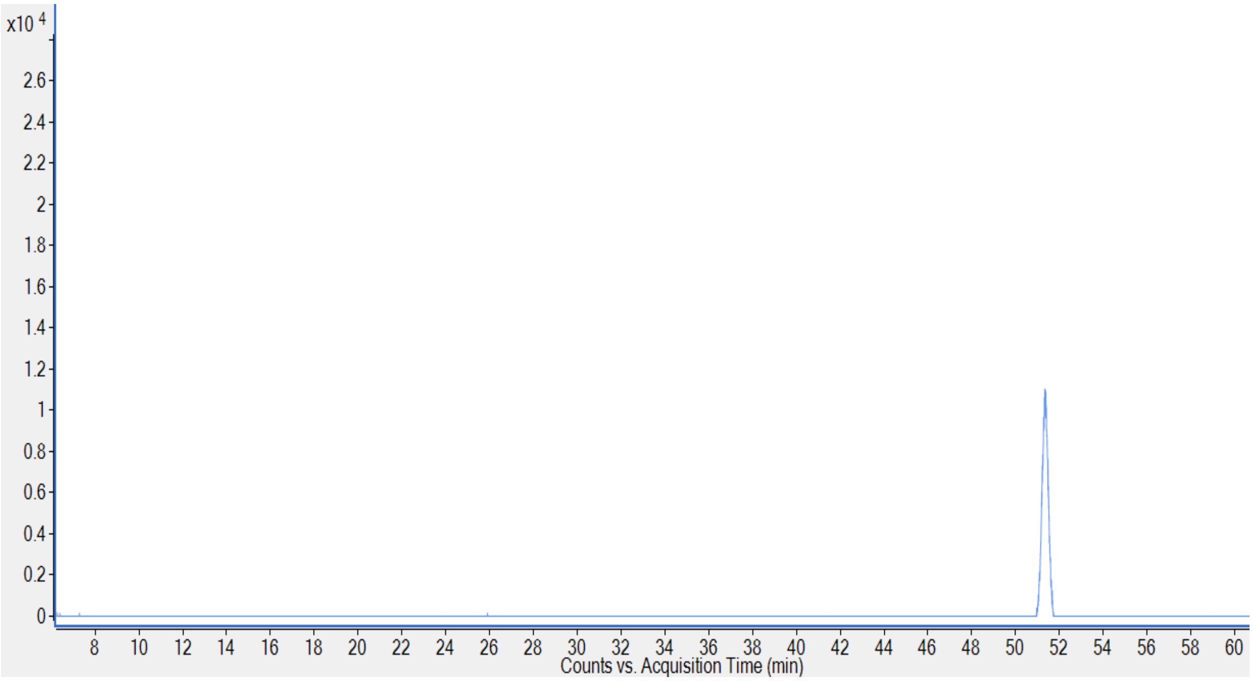

Column: 2,6-dimethyl-3-pentyl-beta-cyclodextrin (50% in polysiloxane), 30 m, 130 °C, pressure: 12.9 psi helium.

7. References

(1) Zhang, W.; Wang, F.; McCann, S. D.; Wang, D.; Chen, P.; Stahl, S. S.; Liu, G. Enantioselective Cyanation of Benzylic C–H Bonds via Copper-Catalyzed Radical Relay. *Science* **2016**, *353*, 1014–1018.

(2) Lai, X.-L.; Chen, M.; Wang, Y.; Song, J.; Xu, H.-C. Photoelectrochemical Asymmetric Catalysis Enables Direct and Enantioselective Decarboxylative Cyanation. *J. Am. Chem. Soc.* **2022**, *144*, 20201–20206.
